# Supplementary material for: Extreme vulnerability to intruder attacks destabilizes network dynamics
Source: Nat Commun. 2026 Aug 13;17:9716. doi: 10.1038/s41467-026-76342-5 (PMC13562742; doi:10.1038/s41467-026-76342-5)
Supplement: Supplementary file 1 [file 41467_2026_76342_MOESM1_ESM.pdf]

Supplementary Information for the paper
Extreme vulnerability to intruder attacks destabilizes network dynamics

Amirhossein Nazerian,[1] Sahand Tangerami,[2] Malbor Asllani,[3] David Phillips,[1] Hernan Makse,[4] and Francesco Sorrentino[5,1,a)]

[1)] *Department of Mechanical Engineering, University of New Mexico, Albuquerque, NM 87131, USA*

[2)] *Department of Mechanical Engineering, K. N. Toosi University of Technology, Tehran, Iran*

[3)] *Department of Mathematics, Florida State University, 1017 Academic Way, Tallahassee, FL 32306, USA*

[4)] *Levich Institute and Physics Department, City College of New York, New York, NY 10031, USA*

[5)] *Max Planck Institute for the Physics of Complex Systems, 01187 Dresden, Germany*

Supplementary Note 1. ALGEBRAIC CONNECTIVITY f

To motivate the relevance of the algebraic connectivity f, we have the following single-integrator consensus dynamics example. We study the effect of unidirectional agent addition in consensus dynamics through the change in the algebraic connectivity f.

Given a graph with Laplacian matrix L, we set

$$\tilde{L}_{off} = \begin{bmatrix} L & \mathbf{0} \\ \mathbf{0}^\top & 0 \end{bmatrix}, \quad \tilde{L}_{on} = \begin{bmatrix} L + \text{diag}(\boldsymbol{b}_{i^*}) & -\boldsymbol{b}_{i^*} \\ \mathbf{0}^\top & 0 \end{bmatrix}.$$

The Laplacian matrix $\tilde{L}_{off}$ describes a graph in which an adversarial agent is added to a network, but no connection between this agent and the original network is made. The Laplacian matrix $\tilde{L}_{off}$ describes the graph after the unidirectional connection from the adversarial agent to the original network is made. The vector $\boldsymbol{b}_{i^*}$ has all zero entries except for the i^*th, which equals -1.

Consider the consensus problem

$$\dot{\boldsymbol{x}}(t) = -\tilde{L}(t)\boldsymbol{x}(t), \quad \tilde{L}(t) = \begin{cases} \tilde{L}_{off} & 0 \le t < T, \\ \tilde{L}_{on} & t \ge T. \end{cases} \tag{S1}$$

in which the effective Laplacian matrix $\tilde{L}$ switches from $\tilde{L}_{off}$ to $\tilde{L}_{on}$ at $t = T$, i.e., the unidirectional connection from the adversarial agent to the original network is made at time T. This indicates that for $0 \le t < T$, the consensus dynamics is stable, and for $t \ge T$, it becomes unstable.

Supplementary Fig. 1 shows the effect of an adversarial agent addition to a directed tree of 7 nodes. It is clear that the adversarial agent destroys the global consensus, since the individual trajectories $x_i(t)$ do not coincide after the unidirectional connection from the adversarial agent is turned on. Also, the norm of the perturbations

$$\|\delta\boldsymbol{x}(t)\| := \sqrt{\sum_{j=1}^{N}\left(x_j(t) - \frac{1}{N}\sum_{k=1}^{N} x_k(t)\right)^2} \tag{S2}$$

decays when the adversarial connection is off and grows when it is on. The rate at which $\|\delta\boldsymbol{x}(t)\|$ grows directly correlates with the algebraic connectivity f.

Next, we quote some of the properties of the algebraic connectivity of digraphs f from[4]:

1. If the reversal of the digraph does not contain a spanning directed tree, then $f \le 0$.
2. Let i and j denote two nodes that are not connected, i.e., $L_{ij} = L_{ji} = 0$. Then $f \le 1/2(\deg_i^{out} + \deg_j^{out})$, where $\deg_i^{out}$ denotes the outdegree of node i. Thus, if two nodes are in the digraph with zero outdegrees, then $f \le 0$.
3. If the digraph is not weakly connected, then $f \le 0$.
4. Let $\bar{f}$ denote the algebraic connectivity of the complement graph with the Laplacian matrix $\bar{L}$. Then, $f + \bar{f} = N$, where N is the number of nodes.
5. For all balanced digraphs, $f \ge 0$.
6. If the digraph H is constructed from the digraph G by removing k nodes and the adjacent edges, then $f_H \ge f_G - k$.

Remark 1. Unlike the case of undirected graphs, it is possible for a digraph to have a negative indegree and $f > 0$. An example of this is the Laplacian matrix

$$L = \begin{bmatrix} 1.7 & 0 & -1.7 \\ 0 & -0.3 & 0.3 \\ -0.2 & -1.6 & 1.8 \end{bmatrix}$$

that has a negative indegree for its second node, $L_{22} = -0.3 < 0$, while $f = 0.4281 > 0$.

Supplementary Note 2. MULTIPLE ATTACKS

In this section, we discuss the case where multiple intruders attack the network. We start by distinguishing between the two cases of:

a)Electronic mail: fsorrent@unm.edu

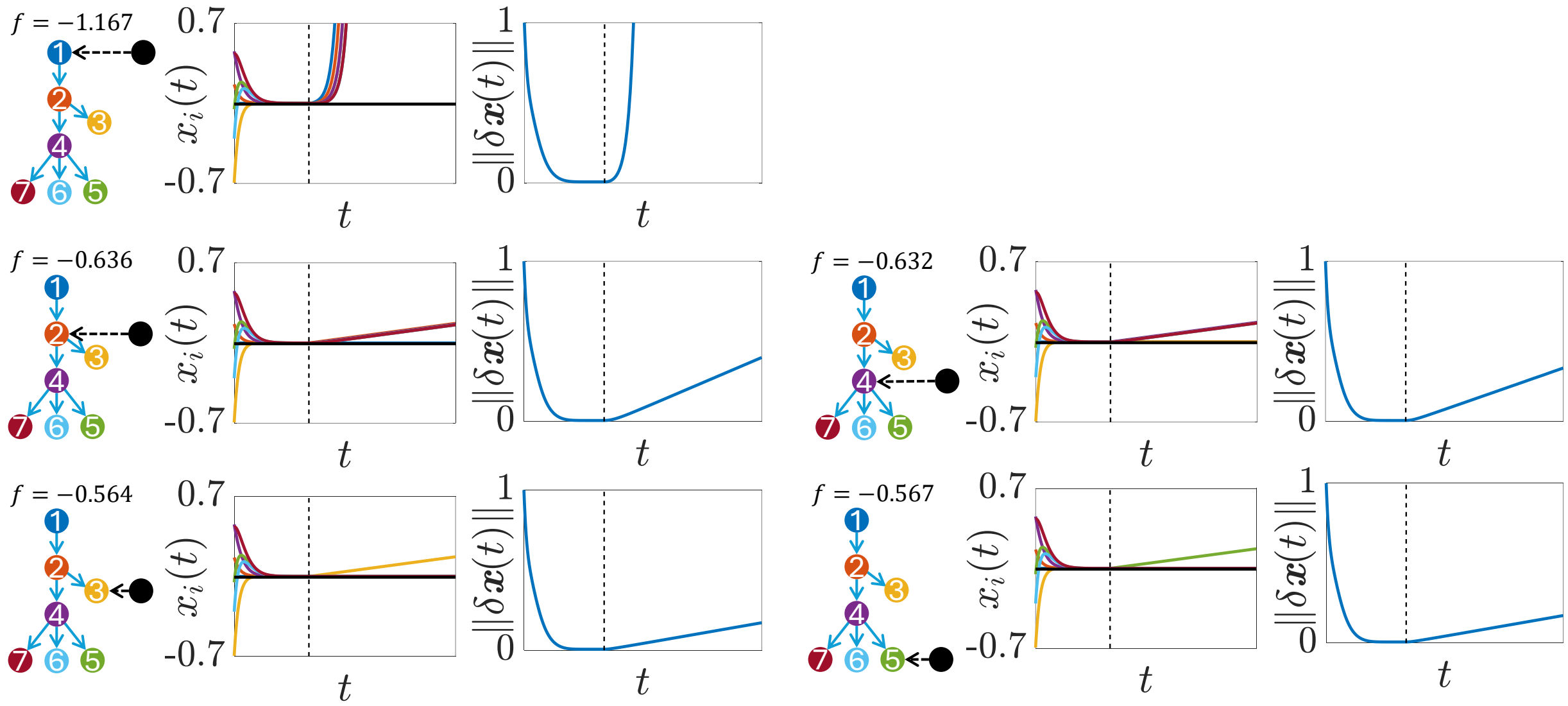

Supplementary Fig. 1. The consensus dynamics as the adversarial agent (black node) is connected to different nodes in the original network. Plots show the individual trajectories of the nodes $x_i(t)$ and the norm of the perturbations about the consensus state $\|\delta \boldsymbol{x}(t)\|$. The dashed black line indicates the instance in which the unidirectional connection from the adversarial agent to the original network is established, i.e., the line corresponds to $t = T$ in Eq. (S1). The algebraic connectivity f is shown for the time the adversarial connection is turned on. The consensus dynamics and the algebraic connectivity f for pinning nodes 6 and 7 are not shown since they are identical to node 5 due to the symmetry of the problem.

1. Sequential multiple attacks.

2. Simultaneous multiple attacks.

In case 1, intruders attack the network sequentially. For this case, our analysis and results already stated in the main manuscript hold, i.e., for each new attack, intruders should target the node with the lowest indegree. The reason is that the optimal first attacks will be on the node with the lowest indegree. After the first attack, the indegree of the attacked node will be reduced by the budget amount, while the indegrees of all other nodes remain intact. As a result, when the second attack is imminent, the attacked node still has the lowest indegree and thus is the optimal candidate for the second attack as well. By induction, all one-by-one attacks should target the node with the lowest indegree to maximize the effect of inducing transient instability.

For case 2, we form an optimization for the simultaneous attacks, inspired by our optimization for a single attack. For simplicity, we consider $m \geq 1$ simultaneous bidirectional attacks on an undirected network with N nodes. Now, the links between the intruders and the original network can be characterized by the matrix $B \in \mathbb{R}^{N\times m}$, where entry $B_{ij} \leq 0$ denotes the strength of the attack from intruder j to node i. Similar to the case of one attack, we set the prescribed budget of the attack to be $-c$, with $c \geq 0$, and require the sum over all the attack links (between the intruders and the network nodes) to be the budget, i.e., $\sum_{i=1}^{N}\sum_{j=1}^{m} B_{ij} = -c$. We aim to find the optimal attack strategy, i.e., the optimal matrix B such that the algebraic connectivity of the following augmented Laplacian matrix is minimized:

$$L^b_{aug} = \begin{bmatrix} L + \mathrm{diag}(B\mathbf{1}_m) & -B \\ -B^\top & \mathrm{diag}(B^\top \mathbf{1}_N) \end{bmatrix}. \tag{S3}$$

Here, $\mathbf{1}_N$ denotes a vector of length N with all entries equal to 1.

Given the symmetric N-dimensional Laplacian matrix L, budget $c > 0$, and the $N \times (N+1)$ matrix $V \in \mathrm{Null}(\mathbf{1}^\top_{N+1})$ with orthonormal columns, we solve for the optimal attack connections $B \in \mathbb{R}^{N\times m}$:

$$f(L^b_{aug}) = \min_{B,\boldsymbol{x}} \quad \boldsymbol{x}^\top \left(V^\top L^b_{aug} V\right) \boldsymbol{x} \tag{S4a}$$

$$\text{s. t.} \quad \boldsymbol{x}^\top \boldsymbol{x} = 1, \tag{S4b}$$

$$L^b_{aug} = \begin{bmatrix} L + \mathrm{diag}(B\mathbf{1}_m) & -B \\ -B^\top & \mathrm{diag}(B^\top \mathbf{1}_N) \end{bmatrix}, \tag{S4c}$$

$$B_{ij} \leq 0, \quad i = 1, \ldots, N, j = 1, \ldots, m, \tag{S4d}$$

$$\sum_{i=1}^{N}\sum_{j=1}^{m} B_{ij} = -c. \tag{S4e}$$

The global solution of the above optimization gives the optimal m simultaneous attack strategy, $B^\star$, that minimizes the algebraic connectivity $f(L^b_{aug}) < 0$, which results in the highest rate of transient instability (minimizing the algebraic connectivity).

We performed extensive numerical simulations where we generated random networks (randomly generated Laplacian matrix L), chose a random budget amount $-c$

and a random number of attackers m, and solved the optimization in Eqs. (S4). Our empirical results show that the optimal attack is to concentrate all the budget $-c$ in one undirected link between any of the intruders and the node with the lowest indegree, i.e., $B^\star = [B^\star_{ij}]$ such that

$$\begin{aligned} &B^\star_{kl} = -c, \quad k = \arg\min_i L_{ii}, \\ &\qquad \text{and } l \text{ is chosen arbitrarily from } 1, \dots, m, \\ &B^\star_{ij} = 0, \quad \forall i \neq k, \forall j \neq l. \end{aligned} \tag{S5}$$

This suggests that only one intruder, say intruder l, should actually attack the network, with all the budget concentrated in a single link toward the node with the lowest indegree, and all others remain disconnected. There is no difference in which intruder is chosen as the attacker (as intruder l), since all intruders are identical before the attack. This finding strengthens our results for a single-intruder attack case by further emphasizing that the optimal attack is to target the node with the lowest indegree and concentrate all budget on a single link. We leave the formal proof for future work and conclude this section with an example.

We take the undirected 7-node network from the power grid example in the main manuscript. Given a budget of $c = 1$, what is the optimal bidirectional attack on the network, given m intruders? Our numerical results suggest that the optimal attack is to concentrate all the budget on a single undirected connection between any of the intruders and the node with the lowest indegree. Figure Supplementary Fig. 2 demonstrates these results by showing that the optimal attack in the case of $m = 1, 2, 3$ intruders is to have only one connection, with all budget concentrated in it. For all cases with $m = 1, 2, 3$, the resulting algebraic connectivity after the optimal attack is $f(L^b_{aug}) = -1.7058$. This indicates the minimum algebraic connectivity $f(L^b_{aug})$ is not a function of the number of intruders m, but a function of the total budget c, which is kept the same for all m. Therefore, the empirical results in Eq. (S5) are confirmed through this example.

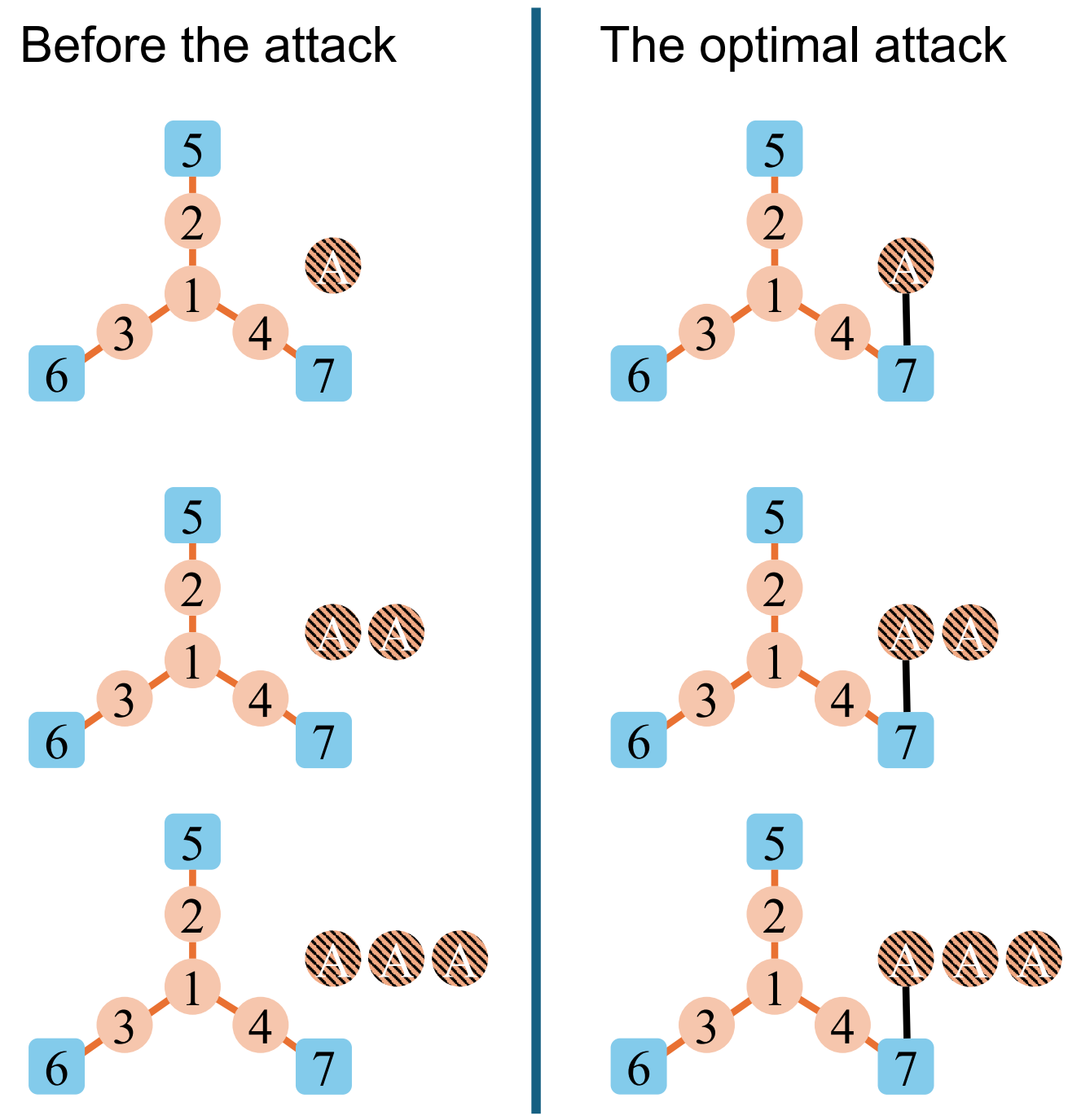

Supplementary Fig. 2. Left panels show the network and the intruder(s) before the attack; right panels show the optimal attack. From top to bottom, the number of intruders is increased, while the total attack budget is kept constant at $c = 1$. The black and orange links have a weight of -1 and 1, respectively. The adversarial agent is shown as node A, while other network nodes are shown in orange (load nodes) and blue (generator nodes) colors.

Supplementary Note 3. ADVERSARIAL AGENT: UNDIRECTED GRAPHS

In this section, we assume that a new agent (node) is being added to the existing network, with the difference that the sign of the connections between the new agent (node) and the existing agents (nodes) is different from that among the existing agents (nodes). We assume the sum of the weights of the new connections is a given budget.

To motivate the importance of studying the adversarial agent problem, we demonstrate the effect of having mixed signed eigenvalues of the Laplacian matrix via examples of single-integrator consensus dynamics.

Example: The Consensus Dynamics. The state of the system i at time t is denoted as $x_i(t)$ and $\dot{x}_i(t) = -\sum_{j=1}^N L_{ij} x_j$, where the matrix $L = [L_{ij}]$ is a Laplacian matrix, i.e., such that $\sum_j L_{ij} = 0$. In compact form,

$$\dot{\boldsymbol{x}}(t) = -L\boldsymbol{x}(t), \tag{S6}$$

where $\boldsymbol{x}(t) = [x_1(t) \;\; x_2(t) \;\; \cdots \;\; x_N(t)]^\top$.

We randomly generate the Laplacian matrix L and add an adversarial node with budget $c = -1$ to the node with the minimum degree to obtain L^b_{aug}:

$$\begin{aligned} L &= \begin{bmatrix} 1 & 0 & -1 & 0 & 0 \\ 0 & 2 & -1 & 0 & -1 \\ -1 & -1 & 3 & -1 & 0 \\ 0 & 0 & -1 & 1 & 0 \\ 0 & -1 & 0 & 0 & 1 \end{bmatrix}, \\ L^b_{aug} &= \begin{bmatrix} 0 & 0 & -1 & 0 & 0 & 1 \\ 0 & 2 & -1 & 0 & -1 & 0 \\ -1 & -1 & 3 & -1 & 0 & 0 \\ 0 & 0 & -1 & 1 & 0 & 0 \\ 0 & -1 & 0 & 0 & 1 & 0 \\ 1 & 0 & 0 & 0 & 0 & -1 \end{bmatrix}. \end{aligned} \tag{S7}$$

The set of eigenvalues of the matrices L and L^b_{aug} is $\Lambda = \{0, 0.51, 1, 2.31, 4.17\}$ and $\Lambda_{new} = \{-1.69, 0, 0.45, 0.83, 2.28, 4.12\}$, respectively. We simulate the consensus dynamics in Eq. (S6) for both

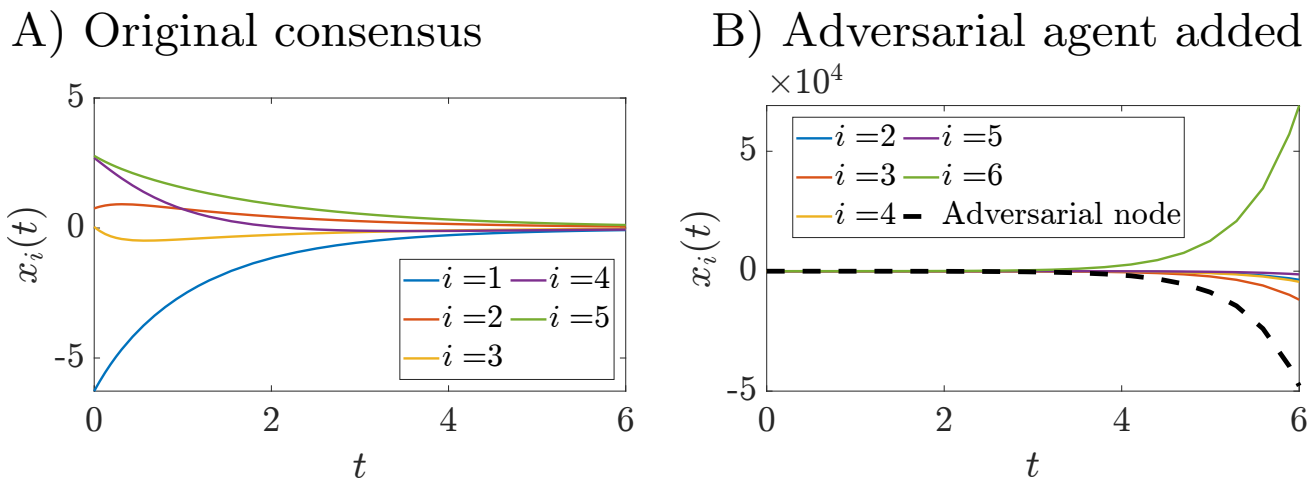

Supplementary Fig. 3. The consensus dynamics in Eq. (S6) for the two Laplacian matrices L and L^b_{aug} from Eq. (S7). Panels A and B show the state trajectories for each system with the Laplacian matrices L (original consensus) and L^b_{aug} (adversarial agent added), respectively. The dashed black line shows the trajectory associated with the newly added adversarial agent.

Laplacian matrices L and L^b_{aug}. The initial conditions are chosen randomly with zero mean as $\boldsymbol{x}(0) = [-6.27, 0.75, 0.04, 2.69, 2.77]$ for the case with L and $\boldsymbol{x}(0) = [-6.2735, 0.7521, 0.0452, 2.6989, 2.7773, 0]$ for the case with L^b_{aug}.

Supplementary Fig. 3A shows the consensus dynamics with the Laplacian matrix L, which is stable, while panel B shows the instability of the dynamics with the Laplacian matrix L^b_{aug}. This difference in stability resulted from having mixed-sign eigenvalues of the Laplacian matrix L^b_{aug} due to the addition of the adversarial agent.

Now we formally introduce the adversarial agent problem:

Problem Statement 3: Given the N-dimensional symmetric Laplacian matrix $L = L^\top \succeq 0$ and a budget $-c \leq 0$, find the weights $\boldsymbol{b} = [b_i]$, $b_i \leq 0$, $i = 1, \ldots, N$, with $\sum_i b_i = -c$ such that the minimum eigenvalue of the matrix

$$L^b_{aug} = \begin{bmatrix} L + \text{diag}(\boldsymbol{b}) & -\boldsymbol{b} \\ -\boldsymbol{b}^\top & -c \end{bmatrix} \tag{S8}$$

is minimized.

Proposition S1. *Given $-c \leq 0$, the minimum eigenvalue of the matrix L^b_{aug}, $\lambda_{\min}(L^b_{aug})$, is bounded as $-2c \leq \lambda_{\min}(L^b_{aug}) \leq -c$.*

Proof. We first prove the upper bound $\lambda_{\min}(L^b_{aug}) < -c$. For all vectors $\boldsymbol{x}$ of norm 1, it follows $\lambda_{\min}(L^b_{aug}) \leq \boldsymbol{x}^\top L^b_{aug}\boldsymbol{x}$. By setting $\boldsymbol{x}_1 = [0 \ \ldots \ 0 \ 1]^\top$, it results $\boldsymbol{x}_1^\top L^b_{aug}\boldsymbol{x}_1 = -c \leq 0$. It thus follows $\lambda_{\min}(L^b_{aug}) \leq -c$ where the equality is satisfied if $\boldsymbol{x}_1$ is an eigenvector which only occurs if $c = 0$ and $\boldsymbol{b} = \boldsymbol{0}$.

Now we prove the lower bound. Based on the Gershgorin circle theorem, at least one of the eigenvalues of the matrix L^b_{aug} lies in the disk centered at $-c$ with radius c. Since we know $\lambda_{\min}(L^b_{aug}) \leq -c$ from above, it thus follows $-2c \leq \lambda_{\min}(L^b_{aug}) \leq -c \leq 0$. □

Proposition S2. *Given $-c \leq 0$, the minimum eigenvalue of the matrix L^b_{aug}, $\lambda_{\min}(L^b_{aug})$, is achieved when $b_{i^*} = -c$ and $b_i = 0$, $\forall i \neq i^*$, i.e., all the budget must focus on one connection.*

Proof. Given $L = L^\top \succeq 0$ and $-c < 0$, we solve

$$\min_{\boldsymbol{b},\boldsymbol{x}} \quad \boldsymbol{x}^\top L^b_{aug}\boldsymbol{x} \tag{S9a}$$

$$\text{subject to} \quad \boldsymbol{x}^\top\boldsymbol{x} = 1, \tag{S9b}$$

$$L^b_{aug} = \begin{bmatrix} L + \text{diag}(\boldsymbol{b}) & -\boldsymbol{b} \\ -\boldsymbol{b}^\top & -c \end{bmatrix}, \tag{S9c}$$

$$b_i \leq 0, \quad \forall i = 1, \ldots, N, \tag{S9d}$$

$$\sum_{i=1}^{N} b_i = -c. \tag{S9e}$$

In the above optimization, we set $\boldsymbol{x} =: [x_1, \boldsymbol{x}_r^\top]^\top$ and $\boldsymbol{x}_r^\top = [x_2,\ x_3, \ldots, x_n, x_{n+1}]^\top \in \mathbb{R}^n$, i.e., we separate the first entry of the vector $\boldsymbol{x}$ from the remaining entries. We also assume $x_i \neq x_j, \forall i \neq j, i = 2, \ldots, N, j = 2, \ldots, N$. The objective function is

$$\boldsymbol{x}^\top L^b_{aug}\boldsymbol{x} = -x_1^2 c - 2x_1\boldsymbol{x}_r^\top\boldsymbol{b} + \boldsymbol{x}_r^\top L\boldsymbol{x}_r + \boldsymbol{x}_r^\top\text{diag}(\boldsymbol{b})\boldsymbol{x}_r \tag{S10}$$

The two terms $-2x_1\boldsymbol{x}_r^\top\boldsymbol{b} + \boldsymbol{x}_r^\top\text{diag}(\boldsymbol{b})\boldsymbol{x}_r$ in the above equation that explicitly depend on $\boldsymbol{b}$ are written as

$$\begin{aligned} -2x_1\boldsymbol{x}_r^\top\boldsymbol{b} + \boldsymbol{x}_r^\top\text{diag}(\boldsymbol{b})\boldsymbol{x}_r &= \sum_{i=1}^{n} -2x_1x_{i+1}b_i + \sum_{i=1}^{n} x_{i+1}^2 b_i \\ &= \sum_{i=1}^{n} \left(x_{i+1}^2 - 2x_1x_{i+1}\right) b_i \\ &=: \sum_{i=1}^{n} \beta_i b_i \end{aligned} \tag{S11}$$

where $\beta_i = x_{i+1}^2 - 2x_1x_{i+1}$, $i = 1, 2, \ldots, n$. Since these two terms from the objective function depend linearly on $\boldsymbol{b}$ and $\sum_j b_j = -c$ and $b_j \leq 0, \forall j$, thus one focuses all budget on the node with the maximum β_i, i.e., $b_{i^*} = -c$, $b_j = 0$, $\forall j \neq i^*$, and $i^* = \arg\max_i \beta_i$. That concludes the proof. □

Next, we analyze the effect of varying the budget $-c \leq 0$ when the connection from the adversarial node is only made to one of the current nodes of the graph. We implemented matrix perturbation theory with a limit of very small and very large c and obtained:

- For small budget c: The eigenvalue $\lambda_{\min}(L^b_{aug}) = -c$.
- For large budget c: the eigenvalue $\lambda_{\min}(L^b_{aug}) = -2c + L_{ii}/2$.

We have summarized the results of this section in Supplementary Fig. 4. For details of the derivations, see the next subsection.

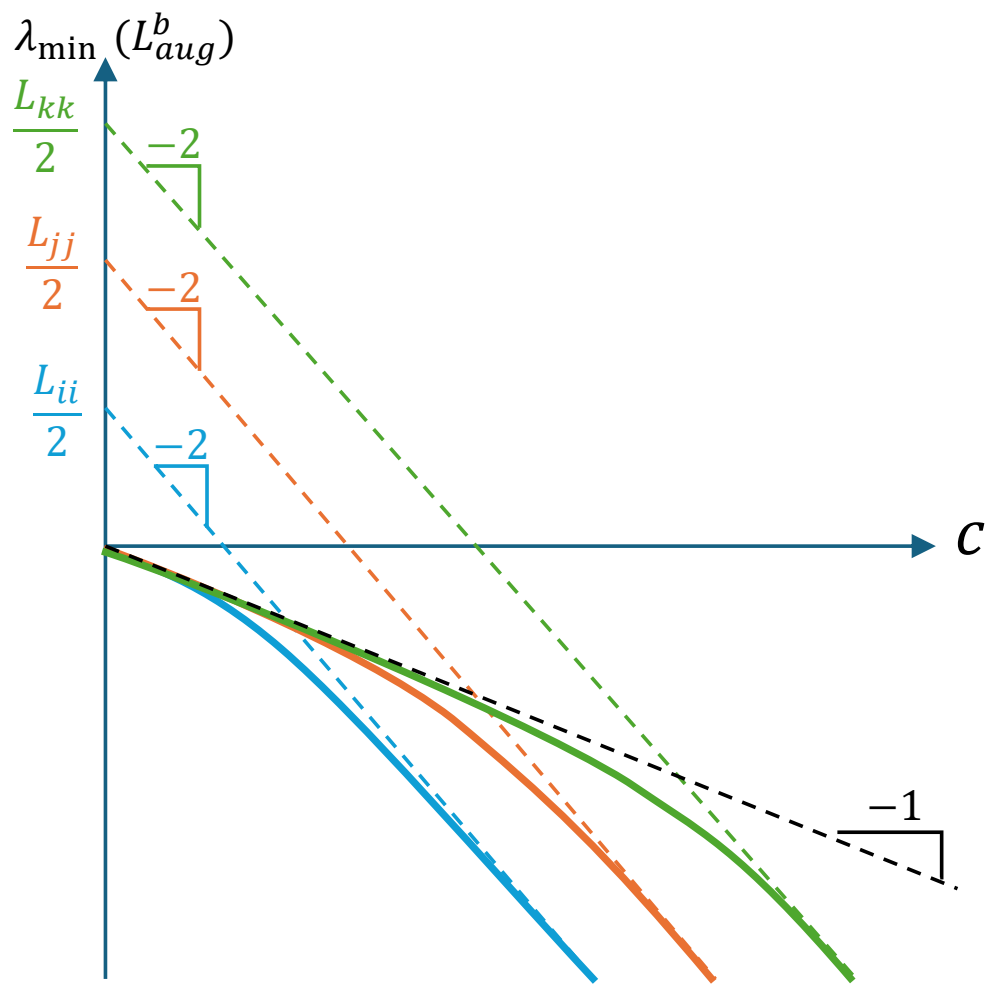

Supplementary Fig. 4. Schematic showing $\lambda_{\min}(L^b_{aug})$, the minimum eigenvalue of the matrix L^b_{aug} in (S8), when only one node (either node i, j, or k) is pinned with the budget $-c < 0$. The dashed black line shows the line $\lambda_{\min}(L^b_{aug}) = -c$. The colored dashed lines show $\lambda_{\min}(L^b_{aug}) = -2c + L_{xx}/2$ for $x = i, j, k$. The schematic is not drawn to scale to enhance visualization.

A. Matrix Perturbation Theory: Undirected Graphs

Here we use matrix perturbation theory to predict the changes in the eigenvalues of a given matrix A_0 subject to a small perturbation ϵA_1, i.e., $\Lambda_\epsilon = \Lambda_0 + \epsilon\Lambda_1 + \epsilon^2\Lambda_2 + \cdots$ where Λ_ϵ is the estimated eigenvalues of the matrix $A_\epsilon = A_0 + \epsilon A_1$, Λ_0 is the exact eigenvalues of A_0, and $\epsilon^k\Lambda_k$, $\forall k \geq 1$, are the kth order approximation of changes in the eigenvalues Λ_0.

In the following, we focus on the case of undirected networks, i.e., for which the matrix A_0 is symmetric, and set $k = 1$. Thus, $\Lambda_1 = \mathrm{diag}(W_0^* A_1 V_0)$, where W_0 and V_0 are the matrices of the left and the right eigenvectors of A_0 such that $W_0^* V_0 = I$, and the superscript * denotes conjugate transpose.

1. **The limit of small c:** In this case, we set $A_0 = \tilde{L}$, $\epsilon = c$, and $A_1 = P_i$ where

$$\tilde{L} = \begin{bmatrix} & & & 0 \\ & & & \vdots \\ & & & 0 \\ & L & & 0 \\ & & & 0 \\ & & & \vdots \\ & & & 0 \\ 0 & \ldots & 0\ 0\ 0\ \ldots\ 0 & 0 \end{bmatrix},$$
$$P_i = \begin{bmatrix} 0 & \ldots & 0 & 0 & 0 & \ldots & 0 & 0 \\ \vdots & \ddots & \vdots & \vdots & \vdots & \ddots & \vdots & \vdots \\ 0 & \ldots & 0 & 0 & 0 & \ldots & 0 & 0 \\ 0 & \ldots & 0 & -1 & 0 & \ldots & 0 & 1 \\ 0 & \ldots & 0 & 0 & 0 & \ldots & 0 & 0 \\ \vdots & \ddots & \vdots & \vdots & \vdots & \ddots & \vdots & \vdots \\ 0 & \ldots & 0 & 0 & 0 & \ldots & 0 & 0 \\ 0 & \ldots & 0 & 1 & 0 & \ldots & 0 & -1 \end{bmatrix} \quad \text{(S12)}$$

where P_i has the entry -1 on the main diagonal in the ith position.

By construction, the matrix $\tilde{L}$ has two zero eigenvalues, one corresponding to the matrix L, and one corresponding to the scalar block at the bottom. We are interested in the change in the zero eigenvalue corresponding to the scalar block. Denoting this eigenvalue as $\lambda_0 = 0$, the first order change in this eigenvalue is $\epsilon \boldsymbol{v}_0^\top A_1 \boldsymbol{v}_0 = c \boldsymbol{v}_0^\top P_i \boldsymbol{v}_0$ where $\boldsymbol{v}$ is the eigenvector of $\tilde{L}$ corresponding to λ_0 which is $\boldsymbol{v}_0 = [0\ 0 \cdots 0\ 1]^\top$. It follows $\boldsymbol{v}_0^\top P_i \boldsymbol{v}_0 = -1$. Therefore, the perturbed eigenvalue after pinning node i is

$$\lambda^i_{\epsilon,0} = \lambda_0 + \epsilon \boldsymbol{v}_0^\top A_1 \boldsymbol{v}_0 = 0 + c \boldsymbol{v}_0^\top P_i \boldsymbol{v}_0 = -c. \quad \text{(S13)}$$

2. **The limit of large c:** Here, we set $A_0 = cP_i$, $\epsilon = 1$, and $A_1 = \tilde{L}$ where the matrices P_i and $\tilde{L}$ are defined in (S12). Now, the eigenvalue of interest is the one and only nonzero eigenvalue of P_i which is $\lambda_0 = -2$ and the corresponding eigenvector is $\boldsymbol{v}_0^i = [0\ \cdots\ 0\ \ -1/\sqrt{2}\ \ 0\ \cdots\ 0\ \ 1/\sqrt{2}]^\top$ where the ith and the last entry of this eigenvectors have nonzero elements. The first order change in the eigenvalue λ_0 is ${\boldsymbol{v}_0^i}^\top A_1 \boldsymbol{v}_0^i = {\boldsymbol{v}_0^i}^\top \tilde{L} \boldsymbol{v}_0^i = L_{ii}/2 > 0$. Therefore, the perturbed eigenvalue after pinning node i is

$$\lambda^i_{\epsilon,0} = \lambda_0 + \epsilon {\boldsymbol{v}_0^i}^\top A_1 \boldsymbol{v}_0^i = -2c + {\boldsymbol{v}_0^i}^\top \tilde{L} \boldsymbol{v}_0^i = -2c + \frac{L_{ii}}{2}. \quad \text{(S14)}$$

We see that λ_ϵ is linearly dependent on c in the limit of small and large c. Therefore, (S13) and (S14) define the asymptotes. The budget at the intersection of the asymptotes defines the cornering budget, which is calculated through $-c_c = -2c_c + L_{ii}/2$ that results in

$$c_c = \frac{L_{ii}}{2}. \quad \text{(S15)}$$

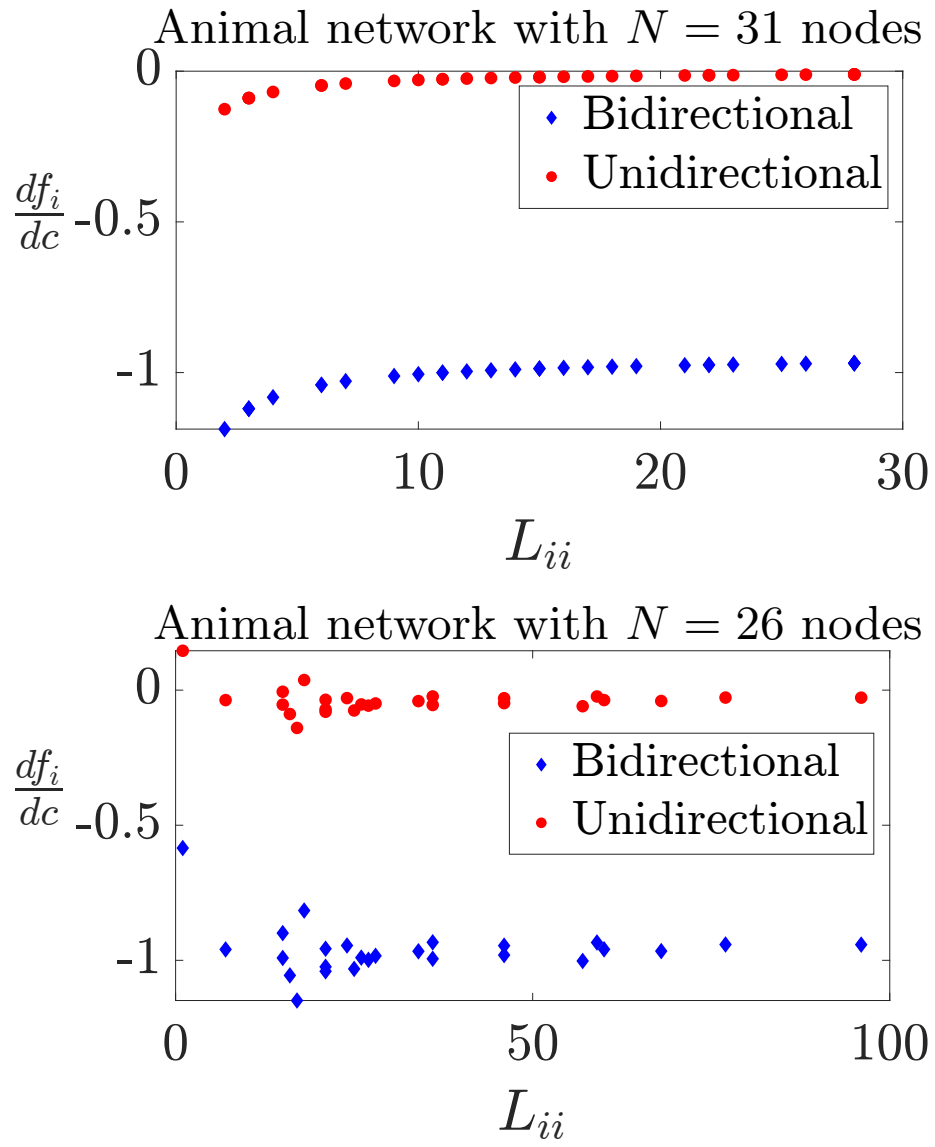

Supplementary Fig. 5. The slope df_i/dc vs. the node indegree L_{ii} for two selected real animal networks with $N = 31$ (top) and $N = 26$ (bottom) nodes, including bidirectional (blue diamonds) and unidirectional (red circles) adversarial connections. The top panel shows a plot in which the slope is directly related to the indegree, while the bottom panel shows a plot in which the slope is not correlated with the indegree.

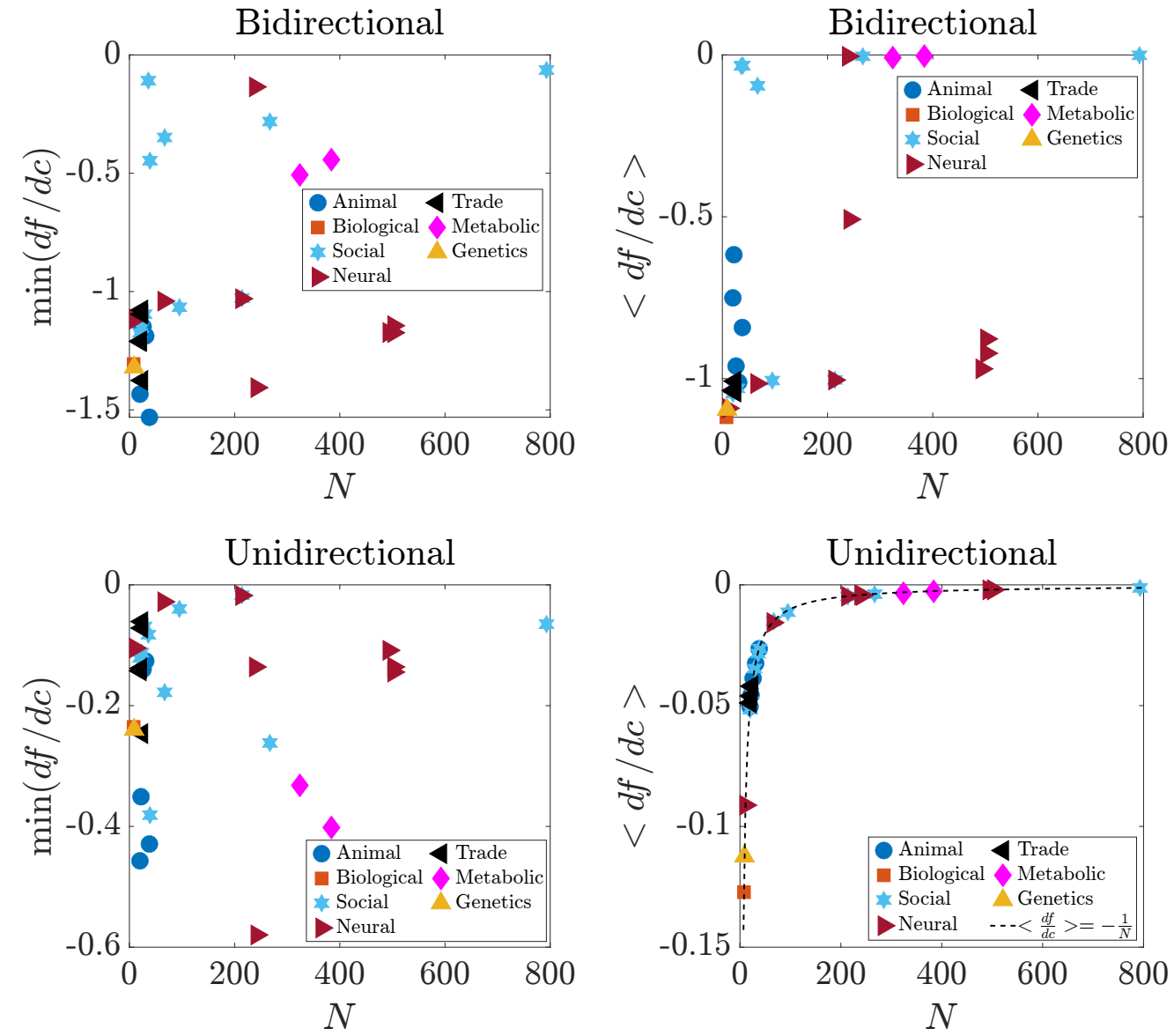

Supplementary Fig. 6. The most negative slope $\min(df/dc)$ and the mean slope $< df/dc >$ for both bidirectional and unidirectional connections of the adversarial agent for selected real networks with size N. The relation between $< df/dc >$ and N in the lower right panel is $< df/dc >= -1/N$ and is shown via the dashed black curve.

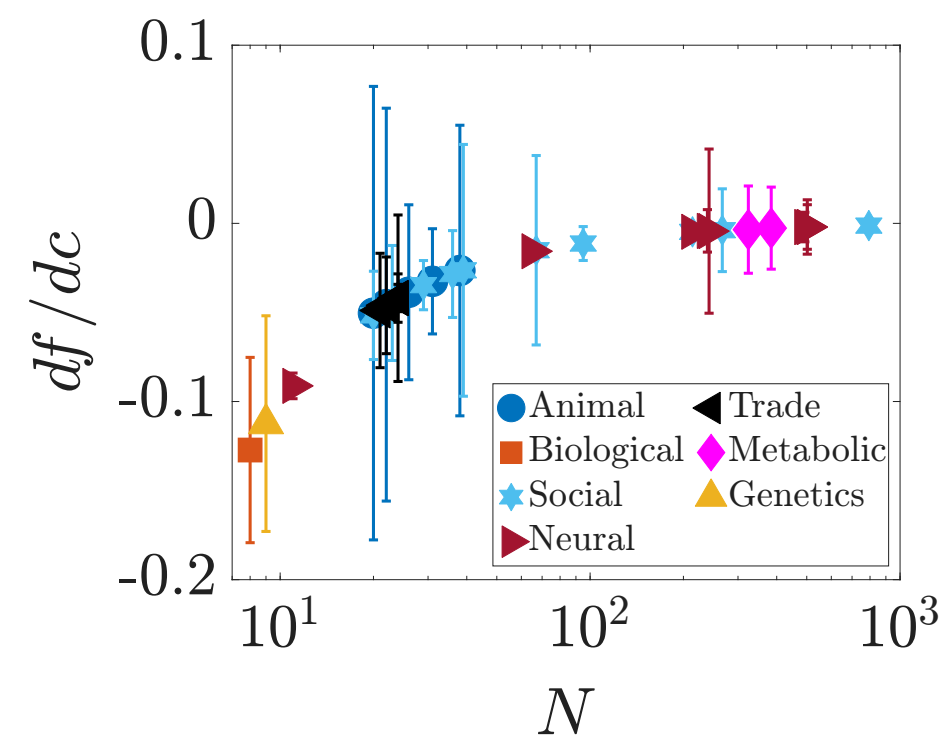

Supplementary Fig. 7. The figure illustrates the case of unidirectional attacks applied to several real-world networks, showing the mean slope df/dc together with its standard deviation (indicated by vertical error bars) as a function of the network size N.

Supplementary Note 4. REAL NETWORKS

First, we emphasize that the slope df_i/dc is not easy to characterize for non-balanced digraphs. This is shown in Supplementary Fig. 5, where the top and bottom panels are plots of the slope df_i/dc versus the indegree of the node i for two selected real networks. In particular, the bottom panel shows a non-monotonic relationship between two quantities.

We calculate the most negative slope $\min(df/dc)$ and the average slope $< df/dc >$:

$$\min\left(\frac{df}{dc}\right) := \min_i \frac{df_i}{dc}, \quad \left\langle \frac{df}{dc} \right\rangle := \frac{1}{N}\sum_{i=1}^{N} \frac{df_i}{dc}. \tag{S16}$$

Supplementary Fig. 6 shows the results of the most negative slope $\min(df/dc)$ and the mean slope $< df/dc >$ for a collection of real networks under bidirectional and unidirectional attacks. The top (bottom) row panels show the results of bidirectional (unidirectional) attacks. The left (right) column panels show $\min(df/dc)$ ($< df/dc >$). It does not seem that the top-row panels and the bottom-left panel show a pattern. Surprisingly, the bottom right panel in Supplementary Fig. 6 shows that the mean slope $< df/dc >$ scales exactly with the size of the network N when the adversarial connection is unidirectional, and the relation is $< df/dc >= -1/N$.

The supplementary information about the real network topologies used in the main manuscript is summarized in Supplementary Table I. The data source is available at[3].

We have also computed the mean slope and its standard deviation for unidirectional attacks applied to several real-world networks. The results are shown in Fig. Supplementary Fig. 7. We observe that, unlike the mean, the standard deviation does not exhibit a correlation with network size.

Finally, we explain the different network-size dependence of the mean slope for unidirectional and bidirectional attacks. To distinguish the two cases, we denote the corresponding unit-budget perturbation matrices by P_i^u and P_i^b, respectively. Let

$$\boldsymbol{y}_0 := V\boldsymbol{v}_0 = \begin{bmatrix} y_{0,1} & \cdots & y_{0,N} & y_{0,N+1} \end{bmatrix}^\top \tag{S17}$$

be the unit-norm effective eigenvector used in the first-order perturbation analysis. Since $\boldsymbol{y}_0$ is in the range of V, it satisfies

$$\boldsymbol{y}_0^\top \boldsymbol{y}_0 = 1, \qquad \sum_{j=1}^{N+1} y_{0,j} = 0. \tag{S18}$$

It follows that

$$\sum_{i=1}^{N} y_{0,i} = -y_{0,N+1}, \qquad \sum_{i=1}^{N} y_{0,i}^2 = 1 - y_{0,N+1}^2. \tag{S19}$$

For a unidirectional attack on node i, first-order perturbation theory gives

$$\begin{aligned} \frac{df_i^u}{dc} &= \boldsymbol{y}_0^\top \frac{P_i^u + (P_i^u)^\top}{2} \boldsymbol{y}_0 \\ &= -y_{0,i}^2 + y_{0,i} y_{0,N+1}. \end{aligned} \tag{S20}$$

Using Eq. (S19), the sum over all possible targeted nodes is

$$\begin{aligned} \sum_{i=1}^{N} \frac{df_i^u}{dc} &= -\sum_{i=1}^{N} y_{0,i}^2 + y_{0,N+1} \sum_{i=1}^{N} y_{0,i} \\ &= -\left(1 - y_{0,N+1}^2\right) - y_{0,N+1}^2 = -1. \end{aligned} \tag{S21}$$

Consequently,

$$\left\langle \frac{df}{dc} \right\rangle^u = \frac{1}{N} \sum_{i=1}^{N} \frac{df_i^u}{dc} = -\frac{1}{N}. \tag{S22}$$

Thus, the small values of the individual unidirectional slopes in Fig. Supplementary Fig. 5 are consistent with the exact $-1/N$ relation.

For a bidirectional attack on node i, the perturbation matrix is symmetric and

$$\begin{aligned} \frac{df_i^b}{dc} &= \boldsymbol{y}_0^\top P_i^b \boldsymbol{y}_0 \\ &= -y_{0,i}^2 + 2 y_{0,i} y_{0,N+1} - y_{0,N+1}^2 \\ &= -\left(y_{0,i} - y_{0,N+1}\right)^2. \end{aligned} \tag{S23}$$

Therefore,

$$\begin{aligned} \sum_{i=1}^{N} \frac{df_i^b}{dc} &= -\sum_{i=1}^{N} \left(y_{0,i} - y_{0,N+1}\right)^2 \\ &= -\sum_{i=1}^{N} y_{0,i}^2 + 2 y_{0,N+1} \sum_{i=1}^{N} y_{0,i} - N y_{0,N+1}^2 \\ &= -\left[1 + (N+1) y_{0,N+1}^2\right], \end{aligned} \tag{S24}$$

and the bidirectional mean slope is

$$\left\langle \frac{df}{dc} \right\rangle^b = -\frac{1 + (N+1) y_{0,N+1}^2}{N}. \tag{S25}$$

Unlike Eq. (S22), Eq. (S25) depends on the component $y_{0,N+1}$ of the effective perturbation eigenvector, which is determined by the network topology. Hence, the bidirectional mean slope does not, in general, reduce to a universal function of the network size N. A formal normalization can be written as

$$\frac{\langle df/dc \rangle^b}{1 + (N+1) y_{0,N+1}^2} = -\frac{1}{N}, \tag{S26}$$

but its normalization factor is network dependent and therefore does not represent a universal network-size scaling law.

In summary, the unidirectional attack admits the topology-independent scaling $\langle df/dc \rangle^u = -1/N$, whereas the bidirectional mean slope retains an explicit dependence on the network topology through $y_{0,N+1}$.

Supplementary Note 5. ADDITIONAL PLOTS AND DETAILS OF THE LORENZ SYNCHRONIZATION EXAMPLE

In this section, supplementary plots for the synchronization example from the main paper are provided, and later on, we provide an explanation of why the synchronization is lost after an attack. Supplementary Fig. 8 shows the $x, y,$ and z components of the coupled Lorenz oscillators before and after the attack. We see the loss of synchronization in all components after the attack occurs.

The stability or instability of the synchronization dynamics can be explained via the Master Stability Function (MSF). The MSF is a plot of the Maximum Lyapunov Exponent of the transverse dynamics to the synchronization manifold as a function of the scalar ν, which can be thought of as any of the nonzero eigenvalues of the Laplacian matrix. If MSF is positive (negative) for any (for all) eigenvalues of the Laplacian matrix, then the synchronization is unstable (stable). The MSF for networked chaotic systems can be categorized into three classes[1] for a non-negative $\nu \geq 0$: Class I for which the value of the MSF is always positive; Class II where the MSF is negative for $\nu > \nu_{\min}$, for some $\nu_{\min} > 0$; Class III where the MSF is negative only in an interval $\nu_{\min} \leq \nu \leq \nu_{\max}$, for some $0 < \nu_{\min} < \nu_{\max}$. For all three classes, the MSF is positive at $\nu = 0$ due to the chaotic nature of the oscillators. When an attack occurs and, as a result, at least one of the eigenvalues of the Laplacian matrix becomes negative, it suggests that the MSF must be studied for $\nu < 0$. For a small budget attack where the magnitude of the negative eigenvalue of the Laplacian is not large, we expect the MSF to remain positive for $\nu < 0$ with small $|\nu|$ due to the continuity, thus resulting in the loss of synchronization.

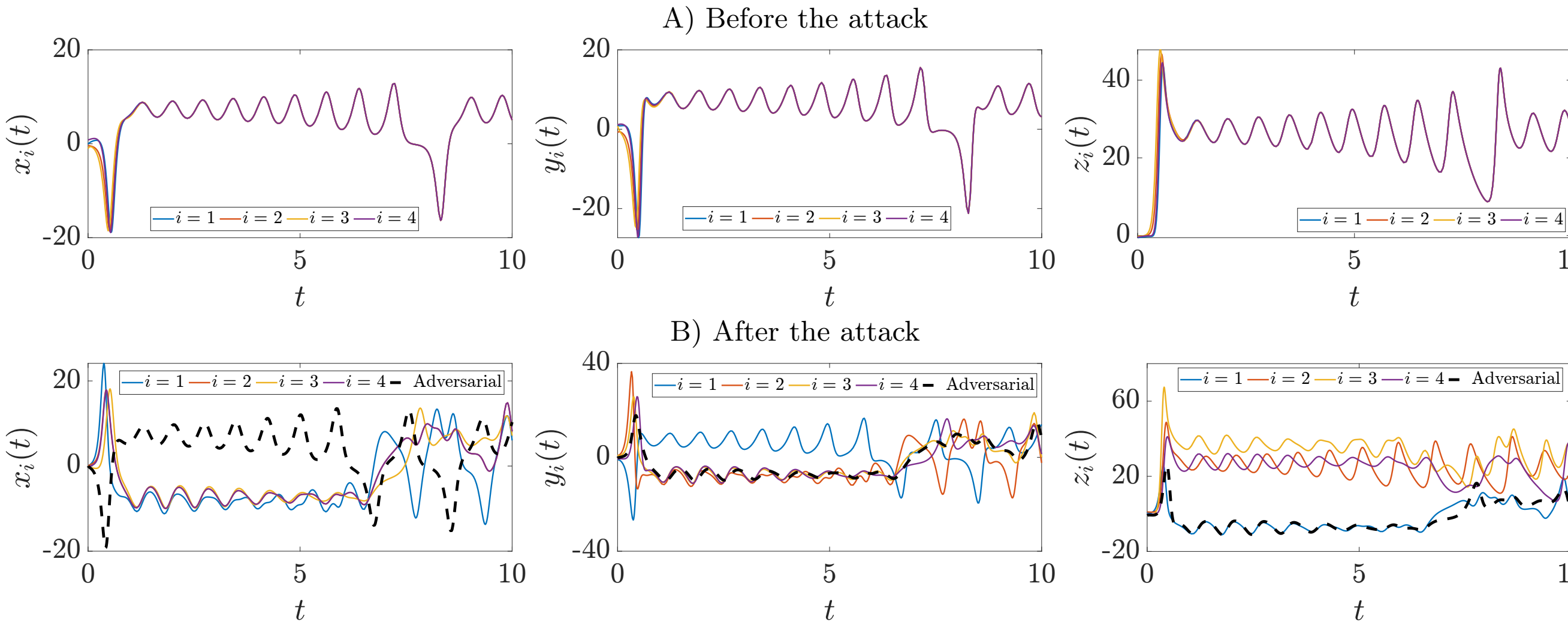

Supplementary Fig. 8. The synchronization dynamics of the Lorenz oscillators for the two Laplacian matrices L and L^b_{aug} from Eq. (4) of the main manuscript. Panels A (top row) and B (bottom row) show the state trajectories for each system with the Laplacian matrices L and L^b_{aug}, respectively. The dashed black line shows the trajectory associated with the newly added adversarial agent.

Supplementary Note 6. FURTHER INFORMATION ON THE CASE OF SYNCHRONIZATION OF COUPLED CHAOTIC OSCILLATORS

In this section, we provide further details on the case study involving synchronization of coupled chaotic oscillators, which is shown in Fig. 5 of the main text. For this example, the state of a single oscillator evolves over time according to the dynamics,

$$\dot{\boldsymbol{x}}_i(t) = \boldsymbol{F}(\boldsymbol{x}_i(t)) - \sigma \sum_{j=1}^{N+1} L_{ij}(t)\boldsymbol{H}(\boldsymbol{x}_j(t)) \tag{S27}$$

$i = 1, \dots, N+1$, where $\boldsymbol{x}_i(t) \in \mathbb{R}^m$ is the state of oscillator i at time t, and the functions $\boldsymbol{F} : \mathbb{R}^m \to \mathbb{R}^m$ and $\boldsymbol{H} : \mathbb{R}^m \to \mathbb{R}^m$ describe the local dynamics and the coupling functions, respectively. The local dynamics $\boldsymbol{F}$ is set to be the chaotic Lorenz oscillator. The functions $\boldsymbol{F}$ and $\boldsymbol{H}$ are

$$\boldsymbol{x} = \begin{bmatrix} x \\ y \\ z \end{bmatrix}, \quad \boldsymbol{F}(\boldsymbol{x}) = \begin{bmatrix} 10(y-x) \\ x(28-z)-y \\ xy-2z \end{bmatrix}, \quad \boldsymbol{H}(\boldsymbol{x}) = \begin{bmatrix} 0 \\ y \\ 0 \end{bmatrix}. \tag{S28}$$

The scalar $\sigma \geq 0$ is the coupling strength, which we set to $\sigma = 10$. The matrix $L(t) = [L_{ij}(t)]$ is the Laplacian matrix that describes the network connectivity and is given by

$$L(t) = \begin{cases} \begin{bmatrix} L_0 & \boldsymbol{0} \\ \boldsymbol{0}^\top & 0 \end{bmatrix}, & t < T, \\ \\ \begin{bmatrix} L_0 & \boldsymbol{b}^i \\ \boldsymbol{0}^\top & 0 \end{bmatrix}, & t \geq T. \end{cases} \tag{S29}$$

Here, $\boldsymbol{b}^i$ is the vector that characterizes the attack from the intruder to node i. We set the attack time to $T = 4s$.

We study the effect of attacking nodes with different indegrees. The choice of targeted nodes is defined by

$$\boldsymbol{b}^i = \begin{bmatrix} 0 & \dots & 0 & -c & 0 & \dots & 0 \end{bmatrix}^\top, \tag{S30}$$

where component i of the vector $\boldsymbol{b}^i$ is equal to $-c$ and all the other components are set to zero. In Fig. 5 of the main manuscript, we keep all parameters and initial conditions fixed and vary i to study the transient instability via the transverse perturbations $\|\delta \boldsymbol{X}(t)\|$.

Supplementary Note 7. FURTHER DISCUSSION ABOUT THE CASE OF THE POWER GRID DYNAMICS

In what follows, we prove that by the addition of an adversarial agent, the dynamics of the power grid as described by the swing equation no longer converge to fixed points. Recalling the swing equation from the main manuscript:

$$\ddot{\vartheta}_i(t) = -\gamma_i \dot{\vartheta}_i(t) + p_i - \sum_{j=1, j\neq i}^{N} A_{ij} \sin(\vartheta_i(t) - \vartheta_j(t)) \tag{S31}$$

where $\vartheta_i(t)$ is the displacement of node i, the scaler p_i denotes the power consumption ($p_i < 0$ for loads) or power generation ($p_i > 0$ for generators,) the scalar $\gamma_i > 0$ denotes the damping ratio, and the symmetric adjacency matrix $A = [A_{ij}]$ describes the connectivity of the grid.

Linearizing Eq. (S31) about the fixed-point ϑ_i^* results in

$$\ddot{\theta}(t) = -\gamma_i \dot{\theta}(t) + p_i - \sum_{j=1}^{N} L_{ij} \theta_j(t) \tag{S32}$$

where $\theta_i(t) := \vartheta_i(t) - \vartheta_i^*$ is the deviations about the fixed-point ϑ_i^* and the matrix $L = [L_{ij}]$ is a symmetric Laplacian matrix where $L_{ij} = \delta_{ij} \sum_{k=1}^{N} A_{ik} \cos(\vartheta_k^* - \vartheta_i^*) - A_{ij} \cos(\vartheta_j^* - \vartheta_i^*)$. Here, δ_{ij} is the Kronecker delta. In what follows, we assume $\gamma = \gamma_i, \forall i$, for simplicity. By defining $\boldsymbol{\theta} := [\theta_1,\ \theta_2, \dots,\ \theta_N]^\top$ and $\boldsymbol{p} := [p_1,\ p_2, \dots,\ p_N]^\top$, the compact form of Eq. (S32) is obtained:

$$\ddot{\boldsymbol{\theta}}(t) = -\gamma \dot{\boldsymbol{\theta}}(t) - L\boldsymbol{\theta}(t) + \boldsymbol{p}. \tag{S33}$$

Since the Laplacian matrix L is symmetric, it can be diagonalized via its eigenvectors. The diagonal matrix $\Lambda = VLV^\top$ has the real eigenvalues $\lambda_1, \lambda_2, \dots, \lambda_N$ of the Laplacian matrix L on its diagonal. The matrix V has the eigenvectors of the Laplacian matrix L as its columns. Assuming the network is connected, there is only one zero eigenvalue, which we denote via $\lambda_1 = 0$, and all other eigenvalues $\lambda_i \neq 0,\ i > 1$. By setting $\boldsymbol{\eta}(t) := V^\top \boldsymbol{\theta}(t)$ and $\boldsymbol{q} := V^\top \boldsymbol{p}$, the coupled compact equation (S33) is then decomposed as

$$\ddot{\boldsymbol{\eta}}(t) = -\gamma \dot{\boldsymbol{\eta}}(t) - \Lambda \boldsymbol{\eta}(t) + \boldsymbol{q}. \tag{S34}$$

The following equation describes the dynamics of each mode of the oscillation

$$\ddot{\eta}_i(t) = -\gamma \dot{\eta}_i(t) - \lambda_i \eta_i(t) + q_i, \quad i = 1, \dots, N. \tag{S35}$$

It is now clear that if any of $\lambda_i < 0$, then the mode $\eta_i(t)$ of the system becomes unstable. Since the overall behavior of the system is the linear combination of the modes, having an unstable mode results in overall instability of the system, i.e., if $\exists i : \eta_i(t) \to \infty$, then $\theta_j(t) \to \infty, \forall j$. Based on Proposition 2 of the main manuscript, we know that if the adversarial agent attacks the system, then we have at least one negative eigenvalue that results in destroying the stability of the linearized swing equation (S32), which in turn suggests that the nonlinear dynamics of the power grid in Eq. (S31) have unstable fixed points.

Supplementary Note 8. ON THE MAGNITUDE OF THE BUDGET

In this section, we comment on what values can be considered 'small' or 'large' for the budget, so that the proper scaling law is used. We perform a numerical study of the 100-node unweighted and undirected scale-free network from Fig. 7 of the main manuscript. We select 5 distinct network nodes to be attacked separately (bidirectional attack). For each attacked node, we vary the budget c and evaluate the algebraic connectivity $f(L^b_{aug})$. Our goal is to see what values of the budget c can be considered as small or large, so that our scaling laws (detailed in Supplementary Note 3) can be applied accordingly:

- For a small budget c: the algebraic connectivity $f(L^b_{aug}) = -c$, and
- For a large budget c: the algebraic connectivity $f(L^b_{aug}) = -2c + L_{ii}/2$.

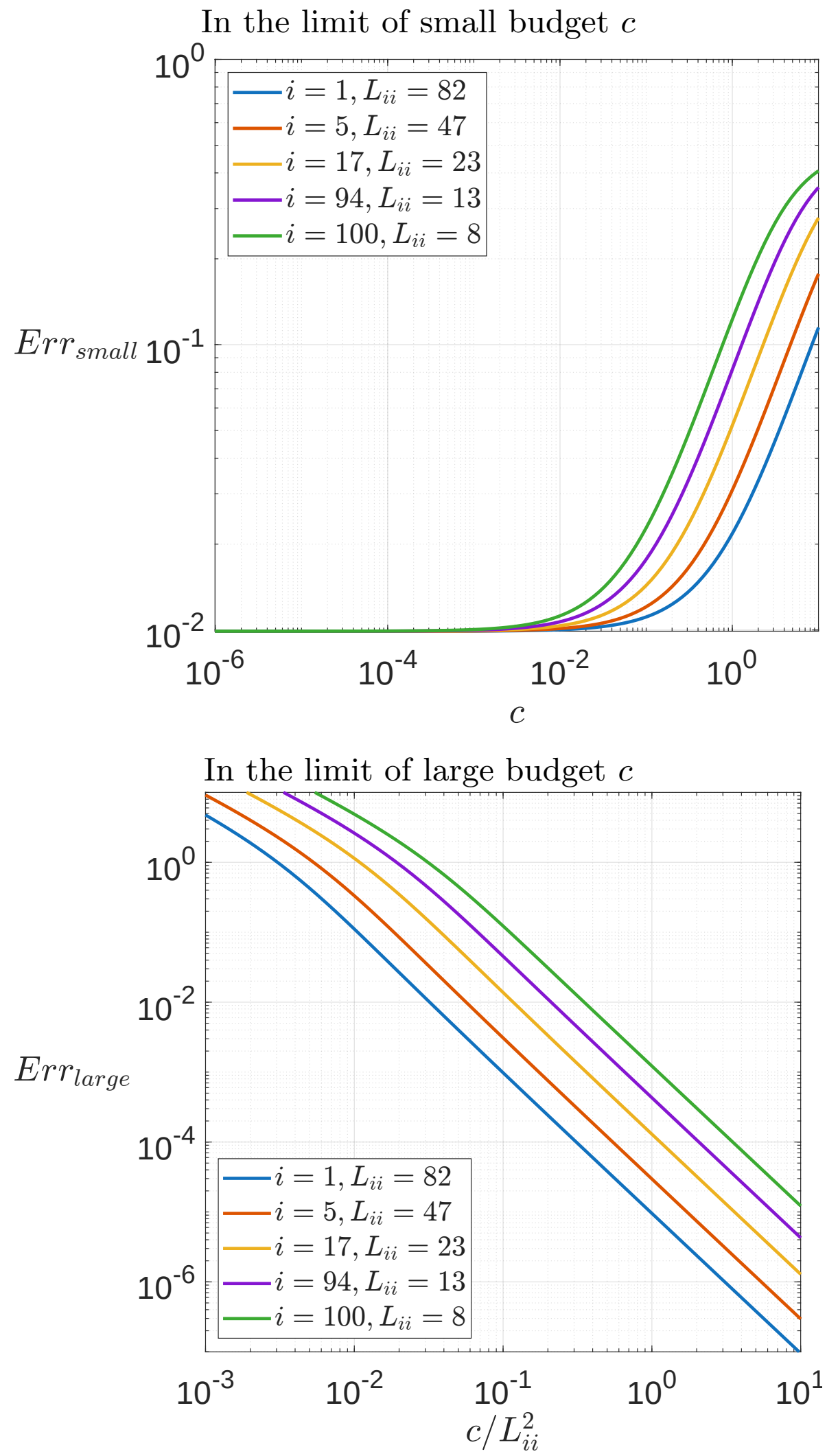

Supplementary Fig. 9. The errors (Eq. (S36)) of our scaling laws in the limit of small (top panel) and large (bottom panel) budget c. The indegree of node i is denoted by L_{ii}.

where L_{ii} denotes the indegree of node i.

To assess the accuracy of the scaling laws above, we define the normalized errors corresponding to small and large values of the budget c, respectively, as:

$$Err_{small} = \left| \frac{f(L^b_{aug}) - (-c)}{f(L^b_{aug})} \right|, \tag{S36a}$$

$$Err_{large} = \left| \frac{f(L^b_{aug}) - (-2c + L_{ii}/2)}{f(L^b_{aug})} \right|. \tag{S36b}$$

We analyze the values of the two errors above to determine the orders in which the budget c can be considered small and large. Supplementary Fig. 9 shows the above errors for small and large values of the budget c. We choose the threshold 10^{-2} for both errors Err_{small} and Err_{large}. We see that we have a low error of the predicted algebraic connectivity $f(L^b_{aug})$; the following bounds for small and large budgets can be considered:

- The small budget should be $c < 10^{-2}$ (independent of the attacked node),
- The large budget should be $c > 0.1 L_{ii}^2$ (dependent on the attacked node).

Supplementary Note 9. EFFECT OF CONNECTIVITY TO THE NETWORK HUB

We randomly generate an 18-node undirected network (left panel in Supplementary Fig. 10), where node 1 has the highest degree ($\deg_1 = 10$), which we refer to as the *hub node*. Nodes 17 and 18 have the lowest degree ($\deg_{17} = \deg_{18} = 1$), and are referred to as *leaf nodes*. The difference between these two leaf nodes is that node 18 is directly connected to the hub, whereas node 17 is not.

We perform the same numerical experiment as in the main manuscript in the context of synchronization, where Lorenz oscillators (with the same local dynamics $\mathbf{F}$ and coupling function $\mathbf{H}$ as in the main manuscript) are coupled through this 18-node network. We set the coupling strength to $\sigma = 9$ and the attack budget to $c = 1$.

As shown in Supplementary Fig. 10 (right panel), the rate of transverse instability of the synchronization dynamics is identical when either node 17 or node 18 is attacked. In both cases, the algebraic connectivity after the attack is $f = -1.6$.

This indicates that vulnerability to intruder attacks is determined solely by the degree of the attacked node, and not by its neighbors, since the change in algebraic connectivity depends only on the node degree (or indegree in directed graphs), as discussed extensively in the main manuscript.

We repeat the above experiment with another randomly generated unweighted and undirected scale-free network. We use the algorithm from[2] to generate a network with 200 nodes. The algorithm generates a network whose degree distribution follows a power-law $P(k) \sim k^{-\gamma}$, where k is the degree. We set the average degree to 20 and the parameter α that controls the power-law to $\alpha = 1$, so that the power-law is $\gamma = (1+\alpha)/\alpha = 2$. The generated network along with its degree distribution is shown in Supplementary Fig. 11 top panels. Node 1, the hub, has the highest degree ($\deg_1 = 195$), and nodes 199 and 200, the leaf nodes, have the lowest degrees $\deg_{199} = \deg_{200} = 1$. Node 199 is connected to the hub, while node 200 is connected to node 160, which has the degree $\deg_{160} = 14$. As shown in Supplementary Fig. 11 (bottom panel), the rate of transverse instability of the synchronization dynamics is identical when either the leaf node 199 (connected to the hub) or the leaf node 200 (connected to a low-degree node) is attacked.

REFERENCES

[1] S. Boccaletti, V. Latora, Y. Moreno, M. Chavez, and D.-U. Hwang. Complex networks: Structure and dynamics. *Physics reports*, 424(4-5):175–308, 2006.

[2] K.-I. Goh, B. Kahng, and D. Kim. Universal Behavior of Load Distribution in Scale-Free Networks. *Physical Review Letters*, 87(27):278701, dec 2001. ISSN 0031-9007. doi: 10.1103/PhysRevLett.87.278701.

[3] J. Kunegis. KONECT – The Koblenz Network Collection. In *Proc. Int. Conf. on World Wide Web Companion*, pages 1343–1350, 2013. URL `http://dl.acm.org/citation.cfm?id=2488173`.

[4] C. W. Wu. *Synchronization in complex networks of nonlinear dynamical systems*. World scientific, 2007.

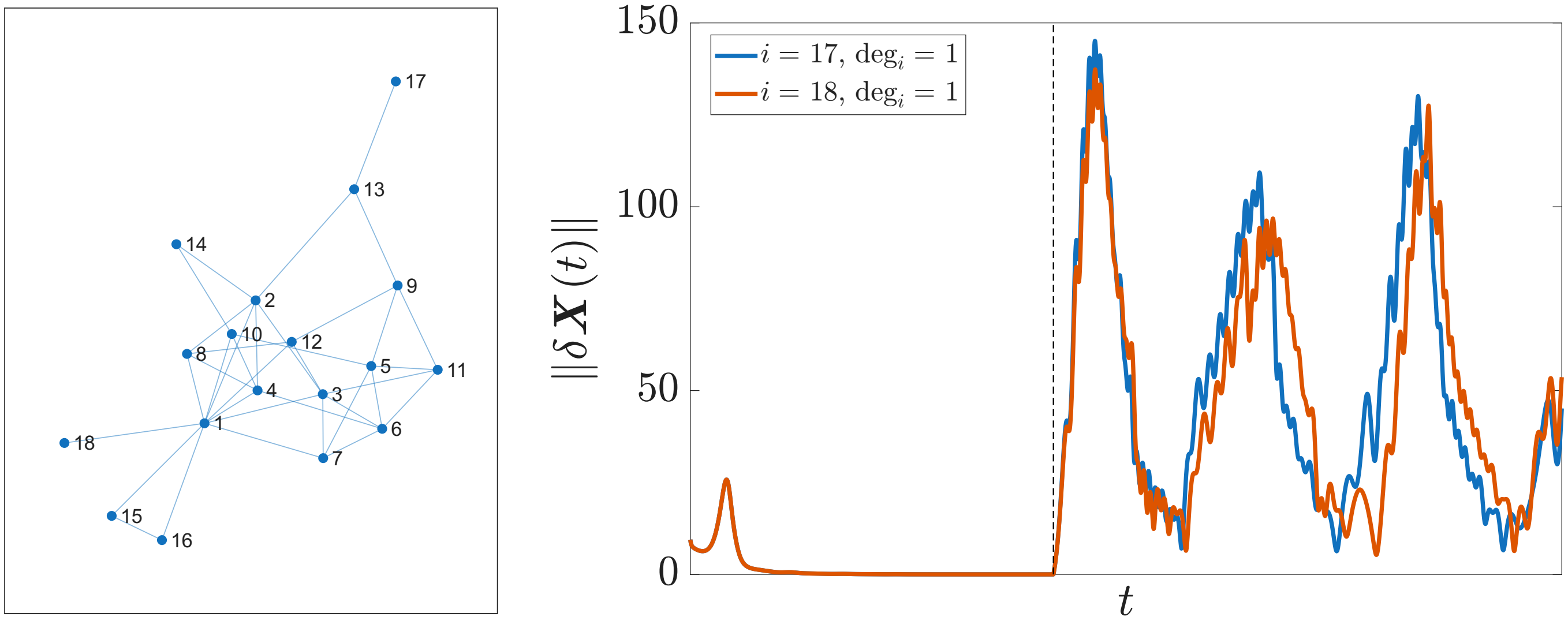

Supplementary Fig. 10. The left panel shows a randomly generated unweighted and undirected network. The right panel shows the norm of the transverse perturbations $\|\delta \boldsymbol{X}(t)\|$ when either one of the leaf nodes 17 and 18 is attacked.

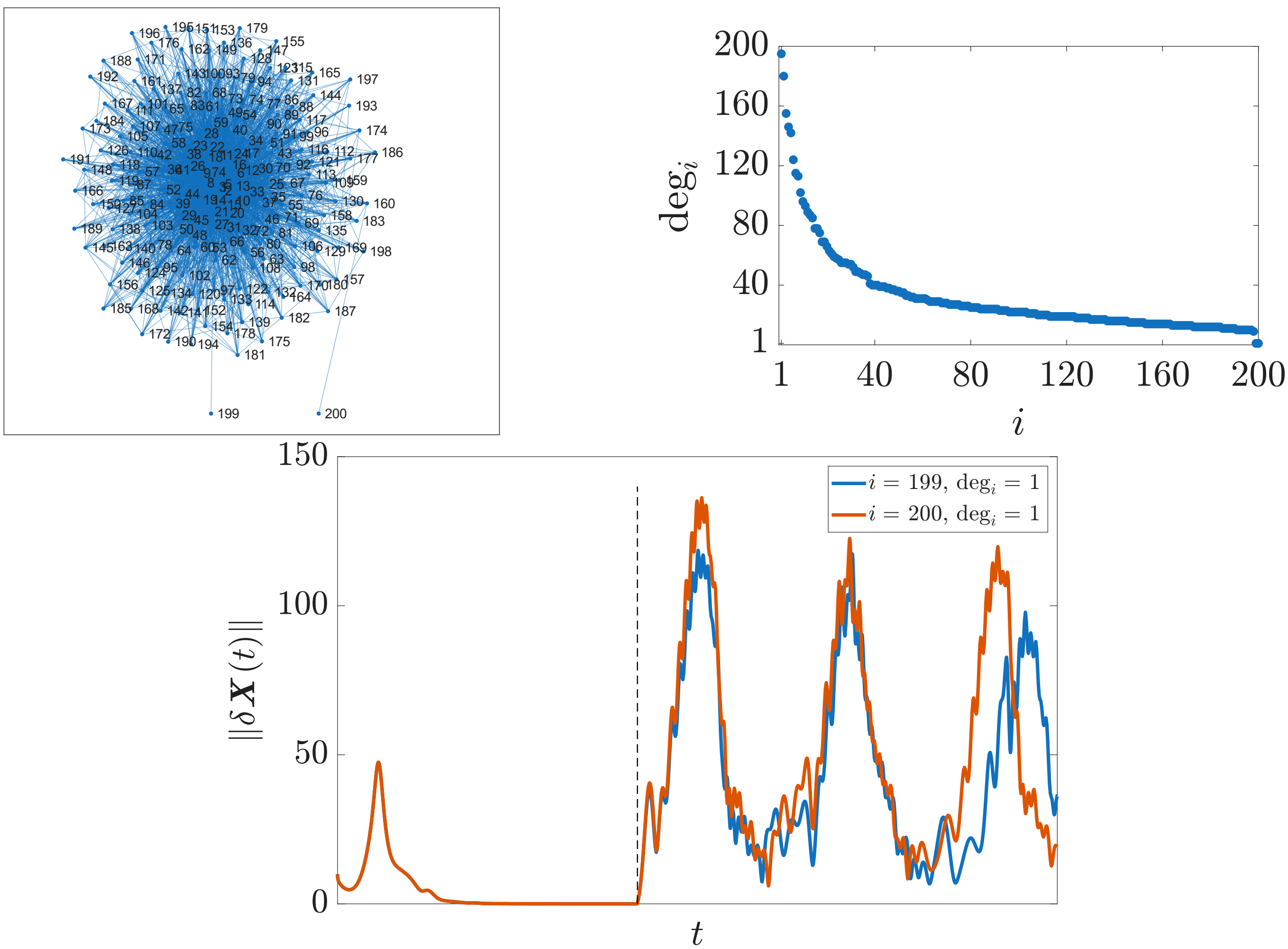

Supplementary Fig. 11. The top left panel shows a randomly generated unweighted and undirected network. The top right panel shows the degree of each node i. The bottom panel shows the norm of the transverse perturbations $\|\delta \boldsymbol{X}(t)\|$ when either one of the leaf nodes 199 and 200 is attacked.

SUPPLEMENTARY TABLE I. **Real Networks**. Detailed description of the real networks considered in the main manuscript. Here, LSCC is short for the largest strongly connected component. The weighted networks are denoted via (w), and the unweighted ones are denoted via (uw.)

Type	Name	Size	LSCC size	Node Info	Edge Info
Animal	Japanese macaques	62	38 (w)	Animal, macaque	Dominance: Only those aggressive interactions were analyzed that led to an outcome in which the loser and the winner could be categorized unambiguously.
	Cattle	28	20 (w)	Animal, mattle	
	Sheep	28	22 (w)	Animal, sheep	
	Bison	26	26 (w)	Animal, bison	
	Hens	32	31 (uw)	Animal, hens	
Biological	Human-protein-interactions (Figeys)	2239	8 (w)	Human, proteins	Protein Interactions.
Social	Social-filmtrust-trust	874	267 (uw)	Human, user	Interactions: Social relationships and communication patterns within different social contexts.
	Social-physicians-trust	241	95 (uw)	Human, physician	
	Social-residencehall-friendship	217	214 (w)	Human, person	
	Social-seventhgraders-proximity	29	29 (w)	Human, student	
	Social-highschool-friendship	70	67 (w)	Human, boy	
Metabolic	net CE	1222	1079 (uw)	Metabolites	The proteins/enzymes interaction.
	Net CQ	436	324 (uw)		
	Net CT	493	384 (uw)		
	Net MJ	1089	1003 (uw)		
	Net SC	1559	1414 (uw)		
Neural	Cat brain	65	65 (uw)	Neurons, neuronal populations, or regions	Functional connectivity.
	Rat brain 1	503	502 (w)		
	Mouse brain	213	213 (uw)		
	Rat brain 2	503	502 (w)		
	Rat brain 3	496	493 (w)		
	Rhesus brain 1	242	242 (uw)		
	Rhesus brain 2	91	11 (uw)		
Trade	Net trade basic	24	22 (uw)	Country	Trade relationship between any two countries.
	Net trade minerals	24	24 (uw)		
	Net trade crude	24	24 (uw)		
	Net trade food	24	24 (uw)		
	Net trade crude	24	21 (uw)		
Genetics	Human (cancer)	4049	9 (uw)	Genes and regulators	The physical and/or regulatory relationships.
