# Supplementary material for: Extreme vulnerability to intruder attacks destabilizes network dynamics
Source: Nat Commun. 2026 Aug 13;17:9716. doi: 10.1038/s41467-026-76342-5 (PMC13562742; doi:10.1038/s41467-026-76342-5)
Supplement: Supplementary file 2 [file 41467_2026_76342_MOESM2_ESM.pdf]

nature portfolio

Peer Review File

Extreme vulnerability to intruder attacks destabilizes network dynamics

Corresponding Author: Professor Francesco Sorrentino

This file contains all reviewer reports in order by version, followed by all author rebuttals in order by version.

Version 0:

Reviewer comments:

Reviewer #1

(Remarks to the Author)
This works provides an interesting distinction between dynamical and structural vulnerability of complex networks. They show that, for small networks, an intrudes can be more effective in perturbing the dynamics if it targets nodes with lower degree rather than hubs.
They provide a nice theoretical framework and corroborate the theory with simulations for different models.

I think that the paper is interesting and well-written and it can potentially be accepted for publication. I have some concerns on some claims made by the authors and on the presentation of some results, as well as many questions, but I think they can all be addressed in the revision.

_if I understand correctly, the authors say that attacks to nodes with lower degree are more effective, as long as the network is small. Could the authors give some intuition on how to determine the size of the network such that this holds? If this phenomenology were true, it would be something that we would observe in some systems. Can the authors provide some examples?

_I seem to understand that large networks are more robust against dynamical perturbation, which makes sense. I think that this leaves two important point unanswered: 1) can the small networks considered by the authors be modules of a larger network? Would then a modular network be more vulnerable or, by the authors' theory, would the disrupted module act as a "big" node with low degree which could impact the larger network? 2) is there a scaling law on the number of intruder which could disrupt larger networks? If many intruders attack simultaneously, would they still need to attack low degree nodes?

_one very interesting claim is that nodes with lowest degree are what make the network more vulnerable. Yet, in Fig. 1, the intruder attacks also the node with highest degree. Why?

_A few years ago, it was shown that the same network can disply different propagation patters depending on the time scale of the dynamical process https://www.nature.com/articles/s41567-018-0409-0 This also affects how nodes responds to perturbations and, I imagine, to attacks. Does this happen also in the case examined by the authors? If not, why?

_This work highlights the importance of nodes with low degree. I think this reference should also be added to the relevant literature on the topic https://journals.aps.org/prl/abstract/10.1103/PhysRevLett.113.078701

_the Master Stability Function was first developed by Fujisaka and Yamada in this paper , before Pecora and Carroll re-branded it and gave it its actual name. The contribution by Fujisaka and Yamada should be cited together with the one by Pecora and Carroll. This is, unfortunately, a very common oversight https://academic.oup.com/ptp/article/69/1/32/1835380

_can the authors give some intuition of the results of Fig 2?

_for directed graphs, the spectrum of the Laplacian is, in general, complex. Does this play any role?

_the analytical results seem very dependent on the budget of the attack c. If there were multiple attackers would the same theory hold with c total budget of the attacks?

_the Kuramoto model is used but no work by Kuramoto is cited. Please cite either the book of the 1975 conference paper in which the Kuramoto model was introduced.

_In the Conclusions, the intuition given on why low degree nodes are more reactive to intruders than hubs makes sense and I found it interesting and instructive. However, I find a bit difficult to understand (and believe) how a large network can be disrupted by attacking a peripheral node. Indeed, in the introductions the authors say that the network has to be small, but this is not recalled in the conclusions, while it's an important point.

_when introducing the reactivity in the Methods, I think that something should be mentioned about non-normality. This point is relevant because real-world networks are non-normal (not scale-free, which are actually rare). Also, I wonder if the pseudo-spectrum can play any role in facilitating the attack.

_would adding higher-order (i.e., many-body) interactions strengthen the system against intruders? There is a vast literature on the higher-order Kuramoto model, so it may not be too hard to test the authors' theory.

_in Fig 5, what would happen if the intruder is deactivated? would the system go back to synchronization?

Minor comments:

_"i.e." and "e.g." need a comma before and after. Most of them afre correct, but I spotted a few where one or both commas a re missing (e.g., a bit after Eq. 2, et the end of the first column of page 3, etc.).

_in a paper about dynamics, calling "f" the algebraic connectivity is a bit unfortunate, as it's usually associated to the evolution law. I'd suggest to find another letter for that.

_in the literature, the network Laplacian has different definitions, one positive semi-definite (as used by the authors), one negative semi-definite (more accurate, as the continuous diffusion operator is as such). The choice of the authors is, of course, completely legitimate, but somewhere it should be acknowledge that it's not the only possible definition of the Laplacian.

Reviewer #2

(Remarks to the Author)
This paper demonstrates that network dynamics can be destabilized by a single adversarial node, specifically showing that targeting low-degree nodes can achieve destabilization more efficiently than targeting hubs. The theoretical analysis using algebraic connectivity and the Master Stability Function approach, along with numerical simulations and experimental verification, is generally well conducted, and the results are intriguing. However, there are several critical concerns regarding the theoretical foundations and positioning of this work that need to be addressed.

1) The most significant issue concerns the relationship with the authors' own previous work, specifically Sorrentino et al. (2007) on "Controllability of complex networks via pinning" (Reference 59). This earlier paper uses very similar theoretical frameworks, including similar network dynamics, the same MSF approach, and extended Laplacian matrices. However, that study argues for the importance of hub nodes for control, which appears to contradict this study's conclusion about the importance of low-degree nodes for destabilization. While Reference 59 appears in the bibliography and is mentioned briefly in one sentence in the Applications section regarding the master stability function for pinning control, there is no substantive discussion of the theoretical relationship between these two works.

2) The authors might argue that the problem settings are fundamentally different, namely control in the 2007 paper versus destabilization in the current study. However, this distinction does not adequately explain the theoretical discrepancy. The framework in Sorrentino et al. (2007) addresses network controllability via pinning, which is actually a more general problem that naturally encompasses destabilization as a special case. Both studies essentially analyze the stability of network dynamics under external influences through modified nodes, using fundamentally the same mathematical approach of augmented Laplacian matrices and spectral analysis. The fact that one study focuses on achieving control while the other focuses on inducing instability does not, in itself, explain why the same mathematical framework yields opposite conclusions regarding which nodes are most critical. A careful theoretical explanation of how these seemingly contradictory results arise from the same mathematical framework is essential but currently absent.

3) A related concern involves the choice of Laplacian matrix. The 2007 study analyzes both combinatorial and normalized Laplacian matrices, whereas the current study primarily employs the combinatorial Laplacian. This difference may be significant. Other work in the literature, such as Yuan et al. (2016), has shown that results on node vulnerability can depend critically on whether the Laplacian is normalized. Specifically, attacks on hub nodes can be effective when using normalized Laplacian, while attacks on low-degree nodes can be effective when using combinatorial Laplacian. This raises the possibility that the effectiveness of low-degree node attacks demonstrated in this study might fundamentally depend on the choice of Laplacian normalization. The authors should provide results using normalized Laplacian to test whether their conclusions hold under that formulation, explain theoretically why the normalization choice leads to different conclusions,

and discuss under what conditions each result applies. Without this analysis, it is difficult to assess whether the main finding represents a fundamental property of network dynamics or an artifact of the particular mathematical formulation chosen.

4) The characterization of the attack budget parameter c also requires more detailed analysis. The study refers to "small" and "large" budgets in the asymptotic analysis but does not provide quantitative definitions or specific threshold values that distinguish these regimes. It is also unclear how the budget relates to the natural coupling strengths in the network. For instance, are normal edge weights typically of order unity, and if so, what does it mean for the attack budget to be "small" or "large" relative to these values? The analysis would benefit from a discussion of how edge weight heterogeneity affects the conclusions and what constitutes a realistic attack budget in practical systems. These details are important for understanding the practical applicability of the theoretical results.

5) The physical implementation of negative coupling also deserves more concrete discussion. The power grid example provides a clear illustration of how adversarial coupling can be realized through anti-phase oscillations. However, for the other systems mentioned, including consensus dynamics, formation control, and general synchronization problems, it remains unclear how adversarial negative couplings would actually be implemented in practice. What are the implementation constraints? Under what circumstances can an adversarial agent realistically establish such connections? A more thorough treatment of implementation feasibility across different application domains would strengthen the practical relevance of this work.

Reviewer #3

(Remarks to the Author)
Referee report for Paper (NCOMMS-26-012151): Extreme vulnerability to intruder attacks destabilizes network dynamics

In this paper, the authors study how a network can be destabilized by introducing a single adversarial node coupled via adversarial connections. They show the performance across several dynamical systems, including the synchronization of coupled chaotic oscillators, power grid dynamics, and phase synchronization based on Kuramoto dynamics. The main contribution of this work, as stated in the abstract, is that a single low-indegree node induces the greatest instability. The authors develop the theory for linear systems and demonstrate that it also extends to nonlinear systems. However, I have several critical concerns about the current version of the manuscript that prevent its publication.

The novelty of the manuscript remains unclear. There is a substantial body of literature on intruder attacks and adversarial perturbations in networks. The authors claim that their finding challenges the common assumption that hubs are the most critical nodes. However, I am not convinced that this result is particularly surprising, nor that it leads to a fundamentally new characterization of network vulnerability, as suggested. My understanding is as follows, and I would appreciate clarification if it is incorrect. For nodes with large indegree, the effect of an adversarial perturbation may be diluted due to the aggregation of many inputs. In this sense, it is not unexpected that low-indegree nodes could exhibit a stronger marginal impact. It may also be important to consider the role of outdegree, which reflects the extent to which a node influences others; nodes with higher outdegree may induce broader system-level effects. In addition, the authors show that the impact of a single node scales proportionally to 1/N. For linear systems, this behavior is not surprising and appears consistent with standard expectations.

The connection between the theoretical developments in Sections A and B and the application section remains unclear to me, despite the authors' attempt to provide an explanation prior to Section A. The authors present f as a smooth function of c. However, the notion of "network destabilization" is not clearly defined. While several figures illustrate the system states over time before and after introducing the adversarial node, there is no quantitative measure of destabilization provided as a function of c. As a result, it is difficult to understand how the theoretical results relate to the observed dynamics. In particular, it is unclear whether destabilization should be interpreted as a continuous or discontinuous function of c, or how one would formally characterize this transition.

The writing of the manuscript requires substantial improvement. References are not presented in proper order, and the exposition is often difficult to follow. Given that the journal targets a general audience, key concepts should be clearly defined both in text and, where appropriate, mathematically. Terms such as instability, destabilization, and most valuable components of a network are introduced without precise definitions, which makes it difficult to interpret the results.
In Equations (4) and (5), the authors introduce the variables
b and c, but their roles are not immediately clear. In particular, it is not evident how one should choose b under a given budget constraint. Should the values b_i follow a specific distribution? Only later does it become apparent that b_i=−1 (or other value) when the new node connects to node i, and b_i=0 otherwise. More broadly, the logical flow is sometimes difficult to follow. For example, in the second paragraph of the introduction, the authors first distinguish between cascading failures and instability, but then immediately state that the paper focuses on the effects of adversarial connections. The relationship between these concepts is not clearly articulated, leaving the reader uncertain about how the discussion of instability connects to the study of adversarial connections.

The assumptions of the manuscript appear either unrealistic or insufficiently general. In the abstract, the authors state that they address the question of how "one or a few intruders" affect network stability; however, the analysis in the paper focuses exclusively on a single-node perturbation. It would be important to understand how the results extend to cases where a fraction of nodes is perturbed. The authors further claim that a single low-indegree node induces the greatest instability. This

claim would benefit from more extensive validation, for example through simulations across a range of dynamical systems, parameter settings, and network structures. In Equations (4) and (5), the authors consider two extreme cases—bidirectional and unidirectional connections. It would be more informative to examine intermediate or more general configurations, such as a node that points to s nodes and is influenced by r nodes.

There also appears to be an inconsistency in the mathematical treatment. In Equation (7), the norm of the perturbation is computed by summing over all N+1 nodes, whereas in Equations (13) and (18), the summation appears to involve only N nodes. This discrepancy should be clarified.

The figure 1 do not clearly convey the main ideas of the paper. As currently presented, they are difficult to interpret and do not provide sufficient support for the claims being made.

Version 1:

Reviewer comments:

Reviewer #1

(Remarks to the Author)
The authors have addressed some of my concerns and, from their reply, I better understand the main message of their work. Still, I think that the new version of the manuscript hasn't improved as much and I wish that the clarity of their reply could reflect in the manuscript as well.
I still think that this work is relevant and the main result is interesting. I believe that, with some effort in the revision of the manuscript, it can be ready for this journal.

I only have a few comments:

_Because I now understand much better some points (thanks to the reply), I have an additional question. what happens if the node with lowest degree has only one link to the hub? For example, in Fig 5A, if the green node is connected only to the blue node (hub), would an attach on the green node effective or would the hub somehow absorb the attack? I think such points need to be addressed, because the claims of the authors are very strong and general.

_On the same line, I think the issue of the attraction basin needs to be tackled, at least from some angle, and I disagree with the authors that it goes beyond the scope of this work. Again, given the strong claims, it is important to know whether the system can go back to the equilibrium and how quickly once the attack has stopped.

Minor comments:

_in the abstract the authors wrote "an intruder agents"

_pag 2, in the part in blue in the left column: need not "to" spread?

Reviewer #2

(Remarks to the Author)
The authors have responded to my previous comments in a thorough and largely satisfactory manner. I have two remaining minor points for the authors.

Regarding Comments 2.1 and 2.2:
The explanation that adversarial coupling shifts the Laplacian spectrum toward negative values, requiring evaluation of the Master Stability Function at negative arguments, is convincing, and the MSF plots in Supplementary Note 6 provide useful supporting evidence. However, the logical chain connecting these observations to the conclusion that targeting low-indegree nodes produces greater instability is currently spread across the main text and supplementary materials. I recommend adding a concise summary of this reasoning in the main text to improve readability.

Regarding Comment 2.3:
The authors' theoretical position is internally consistent: the main result, namely that attacking the node with the smallest L_{ii} is optimal, holds for a general weighted Laplacian. However, the interpretation that low-indegree nodes are the most vulnerable is contingent on the specific Laplacian formulation adopted. Under a normalized Laplacian, where all diagonal entries are equal, this degree-dependent vulnerability disappears. The manuscript currently presents the vulnerability of low-indegree nodes as a broadly applicable finding throughout the abstract, introduction, and conclusions, without acknowledging this dependence. I recommend that the authors moderate the tone of this claim accordingly and clarify that the degree-dependent interpretation arises specifically from the modeling choices embedded in Eq. (1).

Reviewer #3

(Remarks to the Author)
Referee report for Paper (NCOMMS-26-012151A): Extreme vulnerability to intruder attacks destabilizes network dynamics

I appreciate the authors' efforts to revise the manuscript and address the comments from both the other reviewers and me. However, I have several additional comments regarding the revised manuscript and the response letter.

I understand your spectral analysis about the Laplacian and obtaining the critical low-degree nodes from optimization of the global effect but local analysis. As you know, many studies showed the connections between the network eigenvalue properties and degree/degree distribution. (for example Allesina, Stefano, and Si Tang. "Stability criteria for complex ecosystems." Nature 483.7388 (2012): 205-208.) Therefore, it reasonable to add some controlled experiments about the effect of in- and out- degree on its destabilization.

The statement that there is a "(1/N) decrease in average instability" would be more convincing if accompanied by a characterization of the underlying statistical distribution. In particular, the authors should report the variance or dispersion of node-level effects, since the mean may mask substantial heterogeneity. Why do some low-degree or high-degree nodes exert very different impacts on instability?

In the revised version, the authors define destabilization as a transition from a stable regime to an unstable one. However, the manuscript does not clearly specify what constitutes a stable versus an unstable regime. In control theory and dynamical systems, stability is usually defined relative to an equilibrium point. For a linear system, or a nonlinear system linearized around an equilibrium, local stability requires the eigenvalues of the system matrix or Jacobian to have negative real parts. Therefore, destabilizing means the local stability condition does not satisfy. What you intended to define is more about "desynchronization" or other related terms.

A further concern with the current definition is its treatment of partial synchronization. The authors define the order parameter in Eq. (19), where (\rho = 1) corresponds to complete synchronization and (\rho \approx 0) corresponds to a random or incoherent state. Yet, depending on the system parameters and network topology, the system may settle into partially synchronized states with intermediate values of (\rho), such as 0.9, 0.7, or lower. Under the authors' framework, how should the stability of such partially synchronized states be quantified? These ambiguities suggest that the manuscript needs a formal mathematical definition of destabilization, one that is applicable at least to the specific dynamical systems analyzed in the paper. It is better to be applicable to other similar dynamics not studied here.

Version 2:

Reviewer comments:

Reviewer #1

(Remarks to the Author)
The authors satisfactorily addressed all my concerns. I think this paper is ready to be be published in this journal.

I only have very minor comments.

_at the beginning of Sec I (page 17, first column) one reference didn't pass and there is a "?"

_Fig. 10 in the Supplementary is very interesting. I think the authors could add a few more figures with larger networks to show that this works even when the degree of the hub is much higher (given that real networks can have hubs with degrees of also the order of hundreds). This would further strengthen their claim.

(Remarks on code availability)

Reviewer #2

(Remarks to the Author)
The authors carefully revised the ms according to the reviewers' comments. I believe that the current version of the ms meets the criteria for publication.

(Remarks on code availability)

Reviewer #3

(Remarks to the Author)

Referee report for Paper (NCOMMS-26-012151B): Extreme vulnerability to intruder attacks destabilizes network dynamics

The authors have revised the manuscript and addressed most of my previous comments. Therefore, I only have the last remaining major comment regarding the new results about the detailed analysis of -1/N.

According to Equ. (10) and supplementary fig 5, for unidirectional, <df/dc> = 1/N because df_i/dc is close to -1 for all L_ii, which is independent of network sizes (at least for N=31 and 26). However, for the bidirectional case, df_i/dc is close to 0 for each L_ii. Therefore, the sum of df_i/dc is about -N for the unidirectional case but 0 for the bidirectional case. This is why <df/dc> is not a smooth function of N for the bidirectional case. Cearly, there is some consistent behavior, and some normalization may make them comparable.

It is still not understandable why the standard deviation around 20-40 is the largest in SI figure 7. In the SI figure 6 bottom left, the Neural networks have the lowest min(df/dc) and quite high average values. Then, the standard deviation may be larger, but it is not in SI Figure 7. To avioad any mistakes, I will suggest the authors carefully investigate these.

Minor:
References [10] and [18] are the duplications.

(Remarks on code availability)

Open Access This Peer Review File is licensed under a Creative Commons Attribution 4.0 International License, which permits use, sharing, adaptation, distribution and reproduction in any medium or format, as long as you give appropriate credit to the original author(s) and the source, provide a link to the Creative Commons license, and indicate if changes were made.
In cases where reviewers are anonymous, credit should be given to 'Anonymous Referee' and the source.
The images or other third party material in this Peer Review File are included in the article’s Creative Commons license, unless indicated otherwise in a credit line to the material. If material is not included in the article’s Creative Commons license and your intended use is not permitted by statutory regulation or exceeds the permitted use, you will need to obtain permission directly from the copyright holder.
To view a copy of this license, visit https://creativecommons.org/licenses/by/4.0/

Reviewer 1

Reviewer 1 evaluation:

This works provides an interesting distinction between dynamical and structural vulnerability of complex networks. They show that, for small networks, an intrudes can be more effective in perturbing the dynamics if it targets nodes with lower degree rather than hubs. They provide a nice theoretical framework and corroborate the theory with simulations for different models.

I think that the paper is interesting and well-written and it can potentially be accepted for publication. I have some concerns on some claims made by the authors and on the presentation of some results, as well as many questions, but I think they can all be addressed in the revision.

Answer to Reviewer 1 evaluation:
We thank Reviewer 1 for their positive assessment of our work.

Comment Reviewer 1.1:

if I understand correctly, the authors say that attacks to nodes with lower degree are more effective, as long as the network is small. Could the authors give some intuition on how to determine the size of the network such that this holds? If this phenomenology were true, it would be something that we would observe in some systems. Can the authors provide some examples?

Answer to Reviewer 1.1:
We wish to very much apologize to the Reviewer for the confusion. We now realize some of the language we had used in the original paper was misleading. We hope to clarify things here. Our conclusions are that (1) within a given network attacks to nodes with lower indegree are always more effective than attacks on nodes with larger indegree and (2) when comparing different networks, the average effect of adding a small adversarial connection over all the network nodes $< df/dc >$ is exactly equal to N^{-1}, i.e., it is inversely proportional to the number of nodes. We think of these two results as separate, as (1) pertains to a comparison between different nodes within the same network and (2) pertains to a comparison between different networks. We paid attention to remove any possible source of ambiguity from the paper.

Comment Reviewer 1.2:

I seem to understand that large networks are more robust against dynamical perturbation, which makes sense. I think that this leaves two important point unanswered: 1) can the small networks considered by the authors be modules of a larger network? Would then a modular network be more vulnerable or, by the authors' theory, would the disrupted module act as a "big" node with low degree which could impact the larger network? 2) is there a scaling law on the number of intruder which could disrupt larger networks? If many intruders attack simultaneously, would they still need to attack low degree nodes?

Answer to Reviewer 1.2:
We thank the Reviewer for this insightful comment. The Reviewer is correct that larger networks are, on average, more robust to intruder attacks, i.e., when considering the effect of adding a small adversarial perturbation over all possible target nodes. However, our main result that a single adversarial node can destabilize the network holds irrespective of the network size N. The key difference is that the average rate of destabilization decreases with system size, scaling as $1/N$, yet this remains sufficient to disrupt synchronization.

Regarding point (1), we agree that general intuition indicates that a disrupted module could effectively act as a coarse-grained node for the larger system, but its impact would be mediated by its connectivity to the rest of the network. However, our work applies to entire networks and a full treatment of modular architectures is beyond the scope of this paper, but this represents an interesting direction for future research.

Regarding point (2), we have added Supplementary Note 2 in which we discuss the case of multiple attacks. In summary, we see that still the optimal attack, the one that minimizes the algebraic connectivity of the network, is the attack with all budget concentrated in one link from one of the intruders toward the node with the lowest indegree, and all other intruders remain disconnected. There is no difference on which intruder is chosen as the attacker, since all are identical before the attack. These empirical results are summarized in Remark 1 (on page 10) of the revised manuscript. Below are the details of our analysis, copied from the newly added Supplementary Note 2, where we discuss the case of an attack involving multiple intruders.

We start by distinguishing between the two cases of

1. multiple attacks one-by-one,

2. multiple attacks simultaneously.

In case 1, intruders attack the network sequentially. For this case, our analysis and results already stated in the main manuscript hold, i.e., for each new attack, the intruders should target the node with the lowest indegree. The reason is that the optimal first attacks will be on the node with the lowest indegree. After the first attack, the indegree of the attacked node will be reduced by the budget amount, while the indegrees of all other nodes remain intact. As a result, when the second attack is about to happen, the attacked node still has the lowest indegree, and thus, is the optimal candidate for the second attack as well. By induction, all one-by-one attacks should target the node with the lowest indegree for maximum effect of inducing transient instability.

For case 2, we form an optimization for the simultaneous attacks, inspired by our optimization for a single attack. For simplicity, we consider $m \geq 1$ simultaneous bidirectional attacks on an undirected network with N nodes. Now, the links between the intruders and the original network can be characterized by the matrix $B \in \mathbb{R}^{N\times m}$, where entry $B_{ij} \leq 0$ denotes the strength of the attack from intruder j to node i. Similar to the case of one attack, we set the prescribed budget of the attack to be $-c$, with $c \geq 0$, and require the sum over all the attack links (between the intruders and the network nodes) to be the budget, i.e., $\sum_{i=1}^{N}\sum_{j=1}^{m} B_{ij} = -c$. We aim to find the optimal attack strategy, i.e., the optimal matrix B such that the algebraic connectivity of the following augmented Laplacian matrix is minimized:

$$L^b_{aug} = \begin{bmatrix} L + \operatorname{diag}(B\mathbf{1}_m) & -B \\ -B^\top & \operatorname{diag}(B^\top \mathbf{1}_N) \end{bmatrix}. \tag{1}$$

Here, $\mathbf{1}_N$ denotes a vector of length N with all entries equal to 1.

Given the symmetric N-dimensional Laplacian matrix L, budget $c > 0$, and the $N \times (N+1)$ matrix $V \in \operatorname{Null}(\mathbf{1}_{N+1}^\top)$ with orthonormal columns, we solve for the optimal attack connections $B \in \mathbb{R}^{N\times m}$:

$$f(L^b_{aug}) = \min_{B,\boldsymbol{x}} \quad \boldsymbol{x}^\top \left(V^\top L^b_{aug} V\right) \boldsymbol{x} \tag{2a}$$

$$\text{s. t.} \quad \boldsymbol{x}^\top \boldsymbol{x} = 1, \tag{2b}$$

$$L^b_{aug} = \begin{bmatrix} L + \operatorname{diag}(B\mathbf{1}_m) & -B \\ -B^\top & \operatorname{diag}(B^\top \mathbf{1}_N) \end{bmatrix}, \tag{2c}$$

$$B_{ij} \leq 0, \quad i = 1, \ldots, N, j = 1, \ldots, m, \tag{2d}$$

$$\sum_{i=1}^{N}\sum_{j=1}^{m} B_{ij} = -c. \tag{2e}$$

The global solution of the above optimization gives the optimal m simultaneous attack strategy, $B^\star$, that minimizes the algebraic connectivity $f(L^b_{aug}) < 0$, which results in the highest rate of transient instability (minimizing the algebraic connectivity).

We performed extensive numerical simulations where we generated random networks (randomly generated Laplacian matrix L), chose a random budget amount c and random number of attackers m, and solved the optimization in Eqs. (6). Our empirical results show that the optimal attack is to concentrate all the budget $-c$ in one undirected link between any of the intruders and the node with the lowest indegree, i.e., $B^\star = [B^\star_{ij}]$ such that

$$\begin{aligned} &B^\star_{kl} = -c, \quad k = \arg\min_i L_{ii}, \quad \text{and } l \text{ is chosen arbitrarily from } 1, \ldots, m, \\ &B^\star_{ij} = 0, \quad \forall i \neq k, \forall j \neq l. \end{aligned} \tag{3}$$

This suggests only one intruder, say intruder l, actually should attack the network with all the budget concentrated in one link toward the node with the lowest indegree, and all others remain disconnected. There is no difference on

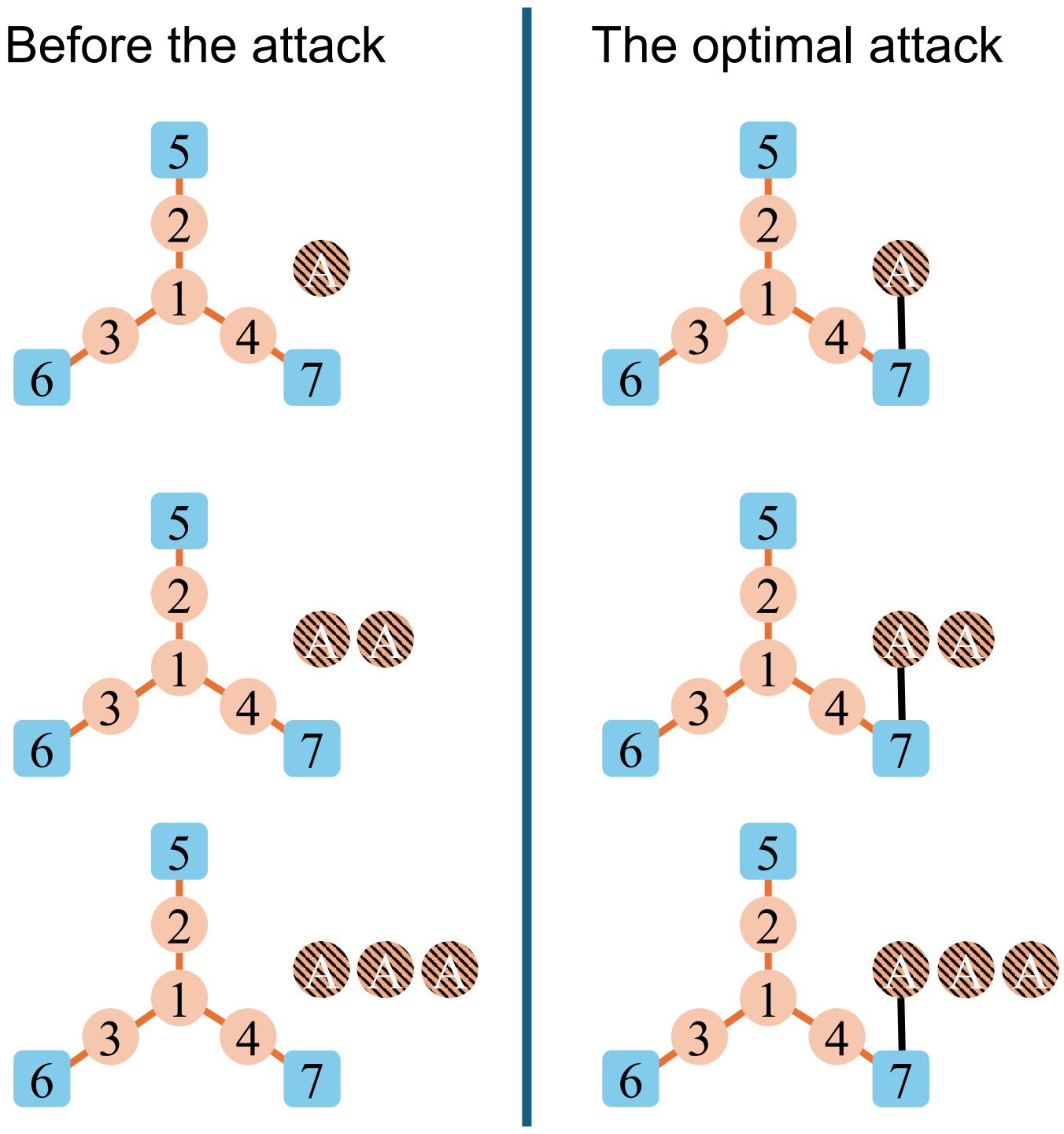

FIG. 1: Left panels show the network and the intruder(s) before the attack; right panels show the optimal attack. From top to bottom, the number of intruders is increased, but the total attack budget is kept the same $c = 1$. The black and orange links have a weight of -1 and 1, respectively. The adversarial agent is shown as node A, while other network nodes are shown in orange (load nodes) and blue (generator nodes) colors.

which intruder is chosen as the attacker (as intruder l), since all intruders are identical before the attack. This finding strengthens our results in the case of one intruder attack by further emphasizing that the optimal attack is to target the node with the lowest indegree and concentrate all budget on one link. We leave the formal proof for future work and conclude this section with an example.

We take the undirected 7-node network from the power grid example in the main manuscript. Given a budget $c = 1$, what is the optimal bidirectional attack on the network, given m intruders? Our numerical results suggest that the optimal attack is to concentrate all the budget in one undirected connection between any of the intruders and the node with the lowest indegree. Figure 1 demonstrates these results by showing that the optimal attack in the case of $m = 1, 2, 3$ intruders is to have only one connection, with all budget concentrated in it. For all cases with $m = 1, 2, 3$, the resulting algebraic connectivity after the optimal attack is $f(L^b_{aug}) = -1.7058$. This indicates the minimum algebraic connectivity $f(L^b_{aug})$ is not a function of the number of intruders m, but a function of the total budget c, which is kept the same for all m. Therefore, the empirical results in Eq. (7) are confirmed through this example.

Comment Reviewer 1.3:

one very interesting claim is that nodes with lowest degree are what make the network more vulnerable. Yet, in Fig. 1, the intruder attacks also the node with highest degree. Why?

Answer to Reviewer 1.3:
We thank the Reviewer for this insightful comment. We agree that the previous version of Fig. 1 could generate confusion regarding the role of node degree in the vulnerability mechanism identified in the paper. For this reason, the figure has been completely redesigned.

Let us first clarify that Fig. 1 is intended to provide an illustration of what we mean by an intruder attack via an adversarial connection; as such, it is primarily pictorial rather than quantitative. In the revised version, the upper panels now depict a simple schematic network containing both a hub node (high indegree) and a peripheral leaf node (zero indegree), together with the possible attachment of the intruder.

Following the Reviewer's suggestion, we now show the intruder connecting to a low-indegree node in Fig. 1B, rather than to a highly connected node as in the original version. This modification more clearly conveys the central analytical result of the manuscript, namely that vulnerability is maximized when the attack targets the node with the smallest indegree, rather than the most connected one. The figure remains purely illustrative and is not used as quantitative evidence; however, it is now fully aligned with the theoretical results and with the notation adopted throughout the paper. We have also revised the labels and caption accordingly to ensure consistency with the rest of the manuscript.

Comment Reviewer 1.4:

A few years ago, it was shown that the same network can disply different propagation patters depending on the time scale of the dynamical process `https://www.nature.com/articles/s41567-018-0409-0` **This also affects how nodes responds to perturbations and, I imagine, to attacks. Does this happen also in the case examined by the authors? If not, why?**

Answer to Reviewer 1.4:
We thank the Reviewer for this insightful comment. The work of Hens et al. shows that the same network topology can display markedly different spatiotemporal propagation patterns when the intrinsic node dynamics is changed, with emphasis on the order and timescale with which nodes respond to a perturbation.

Our paper addresses a different level of description. Rather than analysing the detailed chronology of propagation, we study the instantaneous linear response of the attacked system through spectral measures of the augmented Laplacian or linearized system (Sec. II C). In particular, our analysis is based on the algebraic connectivity and, for directed networks, the complementary reactivity indicator capturing transient growth, which quantifies whether perturbations initially decay or are amplified, and how strongly the attack destabilizes the collective state.

We further show numerically in Figs. 5, 6, and 7, using nonlinear network models, that these instantaneous spectral indicators are strongly consistent with the observed nonlinear response: attack configurations predicted to be more destabilizing according to the linear metric also produce stronger loss of coherence or larger deviations in the full nonlinear dynamics.

Therefore, the central question here is not which node responds first or how the perturbation front travels, but whether the perturbation is amplified once the adversarial connection is introduced. Going beyond these spectral indicators and analysing the full transient or nonlinear post-attack dynamics would be an interesting direction for future work. However, our focus here is centered on a universal spectral criterion for vulnerability: determining whether the network becomes unstable, and which targeted node maximizes that instability.

Comment Reviewer 1.5:

This work highlights the importance of nodes with low degree. I think this reference should also be added to the relevant literature on the topic `https://journals.aps.org/prl/abstract/10.1103/PhysRevLett.113.078701`

Answer to Reviewer 1.5:
We thank the Reviewer for pointing this out. A reference has been added to this paper, which appears to be very relevant.

Comment Reviewer 1.6:

the Master Stability Function was first developed by Fujisaka and Yamada in this paper , before Pecora and Carroll re-branded it and gave it its actual name. The contribution by Fujisaka and Yamada should be cited together with the one by Pecora and Carroll. This is, unfortunately, a very common oversight `https://academic.oup.com/ptp/article/69/1/32/1835380`

Answer to Reviewer 1.6:

We thank the Reviewer for this comment. A reference has been added to the 1983 paper by Fujisaka and Yamada.

Comment Reviewer 1.7:

can the authors give some intuition of the results of Fig 2?

Answer to Reviewer 1.7:
We thank the Reviewer for giving us an opportunity to better present our work. The following text has been added at the end of our presentation of Fig. 2: 'The figure shows that our calculations in the small and large budget limits can be used to sketch the dependence of the algebraic connectivity on c. In both panels, it is shown that the slopes in the small- and large-c limits are constant, while the intercept of the large-c asymptote increases with the node degree. This indicates that the algebraic connectivity is minimized when the node degree is minimal.'

Comment Reviewer 1.8:

for directed graphs, the spectrum of the Laplacian is, in general, complex. Does this play any role?

Answer to Reviewer 1.8:
The Reviewer is correct that the spectrum of a non-symmetric Laplacian is generally complex. In particular, studies [28–32] have shown that a reduction in the imaginary part of the spectrum is often associated with increased non-normality and, consequently, enhanced transient growth. Nevertheless, in our work we focus on the rate of transient instability f, which is, by definition, a real quantity.

Comment Reviewer 1.9:

the analytical results seem very dependent on the budget of the attack c. If there were multiple attackers would the same theory hold with c total budget of the attacks?

Answer to Reviewer 1.9:
We thank the Reviewer for their comment. As addressed earlier, in the case of multiple attacks, we still see that the optimal attack that minimizes the algebraic connectivity is the attack with all the budget c concentrated in one link from one of the intruders toward the node with the lowest indegree, and all other intruders remain disconnected. There is no difference on which intruder is chosen as the attacker, since all are identical before the attack. For more details, we direct the Reviewer to Answer 1.2.

Comment Reviewer 1.10:

the Kuramoto model is used but no work by Kuramoto is cited. Please cite either the book of the 1975 conference paper in which the Kuramoto model was introduced.

Answer to Reviewer 1.10:
We thank the Reviewer for their comment and apologize for the omission. A reference to the book by Kuramoto have been added.

Comment Reviewer 1.11:

In the Conclusions, the intuition given on why low degree nodes are more reactive to intruders than hubs makes sense and I found it interesting and instructive. However, I find a bit difficult to understand (and believe) how a large network can be disrupted by attacking a peripheral node. Indeed, in the introductions the authors say that the network has to be small, but this is not recalled in the conclusions, while it's an important point.

Answer to Reviewer 1.11:
We thank the Reviewer for their comment. We once again apologize for the confusion, which we think was generated by a sentence in the abstract of the original paper. As already mentioned above in our response to Comment 1.2, we have obtained two separate results: (1) within a given network attacks to nodes with lower indegree are always more effective than attacks on nodes with larger indegree and (2) when comparing different networks, the average effect of adding a small adversarial connection over all the network nodes is inversely proportional to the number of nodes. We have now corrected the abstract to avoid any possible confusion between these two separate results.

Although we agree with the Reviewer that this is a counterintuitive and a surprising result, it is indeed possible to destabilize any network, no matter how large it is, by adding a single adversarial connection to any one of its nodes, even one that is peripheral. We find this to be an important message of our paper.

Comment Reviewer 1.12:

when introducing the reactivity in the Methods, I think that something should be mentioned about non-normality. This point is relevant because real-world networks are non-normal (not scale-free, which are actually rare). Also, I wonder if the pseudo-spectrum can play any role in facilitating the attack.

Answer to Reviewer 1.12:
We thank the Reviewer for this insightful comment. The Reviewer is right that the connection between reactivity and non-normality should be stated more explicitly. In the revised manuscript, we have now clarified this point already in the Introduction, where we emphasize that many real-world networks are highly directed, non-normal, and often hierarchically organized, so perturbations may undergo transient amplification and propagate asymmetrically. This provides the structural motivation for introducing reactivity as a complementary indicator to asymptotic stability in directed networks. We have also reiterated this point in the text immediately after the introduction of the algebraic connectivity, where the role of non-normality in generating transient growth is now explicitly stated.

Regarding the pseudospectrum, we agree that it offers a relevant perspective, as the insertion of the intruder has also been considered in our work as a perturbation of the original (pre-attack) network structure, consistent with the framework of pseudospectral analysis. However, pseudospectral analysis is primarily numerical and would not provide the explicit analytical insight achieved by our perturbative approach. In contrast, our method yields clear closed-form conclusions, namely that the optimal strategy is twofold: the full budget should be concentrated on a single target node, and this node is the one with the lowest indegree.

Comment Reviewer 1.13:

would adding higher-order (i.e., many-body) interactions strengthen the system against intruders? There is a vast literature on the higher-order Kuramoto model, so it may not be too hard to test the authors' theory.

Answer to Reviewer 1.13:

We thank the Reviewer for this insightful suggestion. Incorporating higher-order (many-body) interactions is indeed a natural and timely extension of the present framework. In general, higher-order Kuramoto-type couplings can either enhance or hinder synchronization depending on their structure and sign. In particular, cooperative higher-order interactions are known to promote collective coherence and can increase the robustness of the synchronized state, while competing interactions may instead induce frustration, multistability, or cluster synchronization. Relevant references on higher-order synchronization have now been added to the revised manuscript, Refs. [65–69].

We therefore expect higher-order interactions to shift stability thresholds and potentially amplify the role of

localized modes, without altering the underlying mechanism. However, we refrain from making more specific claims, as a systematic analysis of these effects lies beyond the scope of the present work. Exploring this direction in the context of simplicial or hypergraph models would provide a natural extension of our framework and could offer further insight into the robustness of the observed phenomena; we have included a brief discussion of this perspective in the revised manuscript.

Comment Reviewer 1.14:

in Fig 5, what would happen if the intruder is deactivated? would the system go back to synchronization?

Answer to Reviewer 1.14:
We thank the Reviewer for this important question. Yes, the system would go back to synchronization as long as the perturbation remains local about the synchronization manifold (SM). We expect that perturbations on low-indegree nodes have more tendency to result in initial conditions outside the basin of attraction of the synchronized manifold (SM) compared to perturbations on high-indegree nodes. However, it is hard to make a rigorous statement about this as the question of the basin of attraction is notoriously one that is very difficult to address.

Comment Reviewer 1.15:

Minor comments:

"i.e." and "e.g." need a comma before and after. Most of them afre correct, but I spotted a few where one or both commas a re missing (e.g., a bit after Eq. 2, et the end of the first column of page 3, etc.).

Answer to Reviewer 1.15:
We are grateful to the Reviewer for pointing out these typos. We have now fixed them in the revised manuscript.

Comment Reviewer 1.16:

in a paper about dynamics, calling "f" the algebraic connectivity is a bit unfortunate, as it's usually associated to the evolution law. I'd suggest to find another letter for that.

Answer to Reviewer 1.16:
We thank the Reviewer for this thoughtful comment. We understand the Reviewer's viewpoint that in studies of dynamical systems, the letter f is often used to denote the evolution law. However, in our manuscript, this role is consistently assigned to the vector-valued function $\mathbf{F} : \mathbb{R}^m \to \mathbb{R}^m$, while the lowercase f denotes a scalar quantity—namely, the reactivity, which is the central object of our analysis.

Our motivation for using the notation f is that reactivity is consistently treated as a function of the intruder node's budget (i.e., its coupling strength to the network), $f(c)$, throughout the manuscript. Also, to avoid potential confusion, we have carefully checked that this distinction is clearly stated and consistently maintained throughout the manuscript. For these reasons, we prefer to retain the current notation.

Comment Reviewer 1.17:

in the literature, the network Laplacian has different definitions, one positive semi-definite (as used by the authors), one negative semi-definite (more accurate, as the continuous diffusion

operator is as such). The choice of the authors is, of course, completely legitimate, but somewhere it should be acknowledge that it's not the only possible definition of the Laplacian.

Answer to Reviewer 1.17:
We thank the Reviewer for their comment. We have added a sentence to the text where we first introduce the Laplacian matrix (highlighted in blue on page 2) to indicate that another notation is also possible and clarify our particular choice of the notation for the Laplacian matrix.

Reviewer 2

Reviewer 2 evaluation:

This paper demonstrates that network dynamics can be destabilized by a single adversarial node, specifically showing that targeting low-indegree nodes can achieve destabilization more efficiently than targeting hubs. The theoretical analysis using algebraic connectivity and the Master Stability Function approach, along with numerical simulations and experimental verification, is generally well conducted, and the results are intriguing. However, there are several critical concerns regarding the theoretical foundations and positioning of this work that need to be addressed.

Answer to Reviewer 2 evaluation:
We thank Reviewer 2 for reading their careful assessment of the paper.

Comment Reviewer 2.1:

The most significant issue concerns the relationship with the authors' own previous work, specifically Sorrentino et al. (2007) on "Controllability of complex networks via pinning" (Reference 59). This earlier paper uses very similar theoretical frameworks, including similar network dynamics, the same MSF approach, and extended Laplacian matrices. However, that study argues for the importance of hub nodes for control, which appears to contradict this study's conclusion about the importance of low-degree nodes for destabilization. While Reference 59 appears in the bibliography and is mentioned briefly in one sentence in the Applications section regarding the master stability function for pinning control, there is no substantive discussion of the theoretical relationship between these two works.

Answer to Reviewer 2.1:
We thank the Reviewer for their comment. The Reviewer is correct that there are some points in common between this work and the 2007 paper on pinning control. There are, however, two main differences between the two approaches. The first concerns the setting of the problem: in pinning control one considers the role of a reference agent in driving the synchronization of a network of coupled systems toward the reference trajectory, whereas in our work we consider the role of an intruder agent in disrupting synchronization. In the former case the coupling of the reference agent to the rest of the network is cooperative (also sometimes called mutualistic or attractive), while in the latter it is adversarial (also sometimes called competitive or repulsive). In particular, the insertion of an intruder effectively alters the coupling structure so that the relevant Laplacian spectrum is shifted toward negative values; as a result, the asymptotic stability analysis naturally involves the evaluation of the Master Stability Function at negative arguments, corresponding to these eigenvalues. In terms of the Master Stability Function (MSF), this means that one needs to assess the negative values of its argument $K := \sigma\lambda_i$ where λ_i are the eigenvalues of the augmented Laplacian, which is not the common approach in the literature. More importantly, the MSF looks quite different for positive and negative values of its argument (more about this below.) The second difference concerns the observable used to characterize the effect of the intervention: while Sorrentino et al. (2007) focus on asymptotic stability through Lyapunov exponents, here we focus on the transient rate of instability. This distinction is further illustrated by the MSF analysis presented below and in the Supplementary Information. Following the Reviewer's input we have now added an extensive discussion of these points in the revised manuscript. Both these points are important and should be kept in mind when attempting to address the question posed by the Reviewer.

In the revised manuscript, we provide MSF plots associated with Lorenz, Rossler, and Chua's circuit oscillators under various couplings. We direct the Reviewer to the newly added Supplementary Note 6 for details (such as exact local and coupling functions, the linearization, the Maximum Lyapunov Exponent evaluation, etc.). Figure 2 below shows that the MSF is positive in all different coupling setups for the negative range of the MSF argument K. Thus, it shows that the synchronization is locally unstable for negative ranges of the parameter K. We have also made a note of these results in the main manuscript, see the parts in blue in the subsection "The Synchronization of Coupled Chaotic Oscillators". In particular, this sentence has been added at the end of the subsection to relate the results in Fig. 5 with the master stability analysis of Supplementary Note 6:

Note that this is consistent with the MSF analysis for Eq. (12), presented in Supplementary Note 6, since attacking nodes with lower indegrees corresponds to evaluating the master stability function at more negative values of its

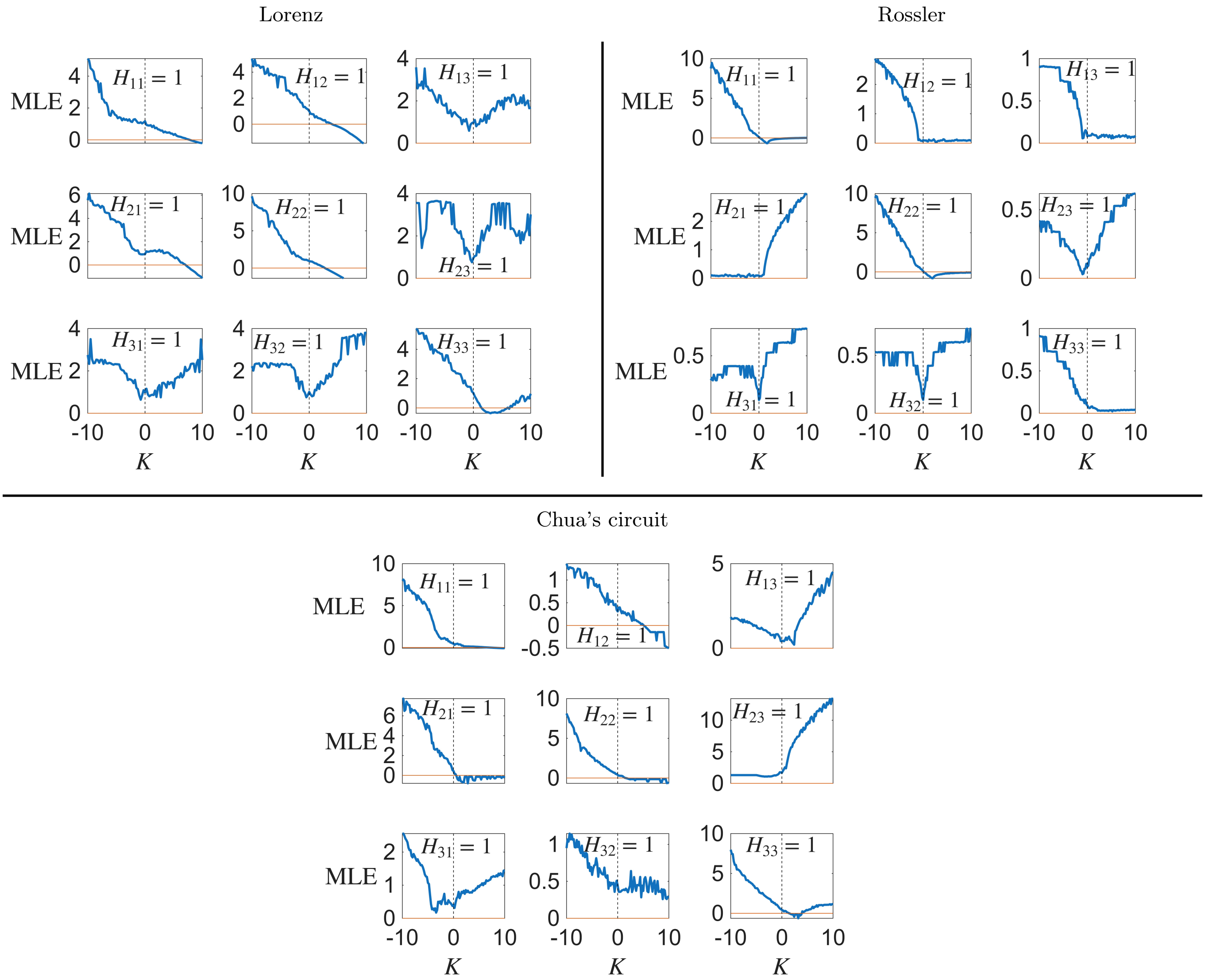

FIG. 2: The master stability plots of the Lorenz system (top left panels), the Rossler system (top right panels), and the Chua's circuit system (bottom panels). Plots show the Maximum Lyapunov Exponent (MLE) as the argument K of the MSF is varied. The coupling is linear as $\boldsymbol{H}(\boldsymbol{x}) = H\boldsymbol{x}$. In each panel, we indicate the only nonzero entry of the matrix H for that case with all other entries of the matrix H set to zero.

argument, where the associated maximum Lyapunov exponent is larger.

Comment Reviewer 2.2:

The authors might argue that the problem settings are fundamentally different, namely control in the 2007 paper versus destabilization in the current study. However, this distinction does not adequately explain the theoretical discrepancy. The framework in Sorrentino et al. (2007) addresses network controllability via pinning, which is actually a more general problem that naturally encompasses destabilization as a special case. Both studies essentially analyze the stability of network dynamics under external influences through modified nodes, using fundamentally the same mathematical approach of augmented Laplacian matrices and spectral analysis. The fact that one study focuses on achieving control while the other focuses on inducing instability does not, in itself, explain why the same mathematical framework yields opposite

conclusions regarding which nodes are most critical. A careful theoretical explanation of how these seemingly contradictory results arise from the same mathematical framework is essential but currently absent.

Answer to Reviewer 2.2:
As already mentioned, we agree that both our work and Sorrentino et al. (2007) rely on a similar mathematical framework based on augmented Laplacians and spectral analysis. The apparent discrepancy does not stem simply from "control vs. destabilization," but from two fundamental differences between the two approaches, see our response to point 2.1 above. In particular, there is a fundamental difference in the sign and role of the coupling introduced by the modified node.

In pinning control, the reference node is cooperatively coupled to the network, effectively adding stabilizing terms to the Laplacian. In practice, this often shifts the spectrum in a direction that enhances synchronization. In contrast, in our setting the intruder node is adversarially (repulsively) coupled, which often induces a qualitatively different spectral effect. Rather than enhancing stability, the perturbation acts to reduce the effective algebraic connectivity. From the perspective of the Master Stability Function (MSF), this corresponds to probing the stability of the synchronized state for negative values of the function argument, whereas pinning control operates in the positive domain. Importantly, the MSF is generally highly asymmetric with respect to the sign of its argument, so that stabilizing and destabilizing perturbations do not mirror each other. The plots in Fig. 2 above show the variety of possible behaviors of the MSF plotted versus its negative and positive argument, from which one can appreciate its variability and lack of symmetry in many cases.

Based on the Reviewer's feedback, we will also attempt to provide a qualitative interpretation for the different behavior that we see in comparison with the 2007 study. In the cooperative case, highly connected nodes tend to be optimal because they distribute stabilization efficiently. In the adversarial case, however, targeting low-degree nodes can be more effective, as these nodes are less constrained by the network and more easily driven away from the synchronized manifold, thereby triggering instability. In other words, the same spectral framework yields different conclusions because the perturbation shifts eigenvalues in opposite directions depending on the sign of the coupling. Thus, the apparent contradiction is resolved by recognizing that the two problems probe different regions of the same stability landscape, leading to distinct optimization criteria for node selection.

Comment Reviewer 2.3:

A related concern involves the choice of Laplacian matrix. The 2007 study analyzes both combinatorial and normalized Laplacian matrices, whereas the current study primarily employs the combinatorial Laplacian. This difference may be significant. Other work in the literature, such as Yuan et al. (2016), has shown that results on node vulnerability can depend critically on whether the Laplacian is normalized. Specifically, attacks on hub nodes can be effective when using normalized Laplacian, while attacks on low-degree nodes can be effective when using combinatorial Laplacian. This raises the possibility that the effectiveness of low-degree node attacks demonstrated in this study might fundamentally depend on the choice of Laplacian normalization. The authors should provide results using normalized Laplacian to test whether their conclusions hold under that formulation, explain theoretically why the normalization choice leads to different conclusions, and discuss under what conditions each result applies. Without this analysis, it is difficult to assess whether the main finding represents a fundamental property of network dynamics or an artifact of the particular mathematical formulation chosen.

Answer to Reviewer 2.3:
We respectfully disagree with the Reviewer on this point. In this work, we consider the formulation presented in Eq. (1) of the main manuscript, in which the coupling between different nodes of the network is described by a Laplacian matrix L. Our result is that under an intrusion attack, the node with the lowest L_{ii} is the optimal node to be attacked. As long as the dynamics are truly described by this equation, our results hold. This result is general, independently of the Laplacian formulation. In the case of a Laplacian matrix with all ones on its main diagonal (e.g., a normalized Laplacian matrix), all nodes are equally vulnerable to being attacked.

We remark that in our paper we work with a generic weighted Laplacian, which covers both the cases of the normalized Laplacian and combinatorial Laplacian as subcases. The only assumption on the Laplacian matrix in our work is that the Laplacian matrix is proper, i.e., all the eigenvalues of L have non-negative real parts, and there is only one eigenvalue equal to 0. None of our results are limited to either normalized Laplacian or combinatorial Laplacian.

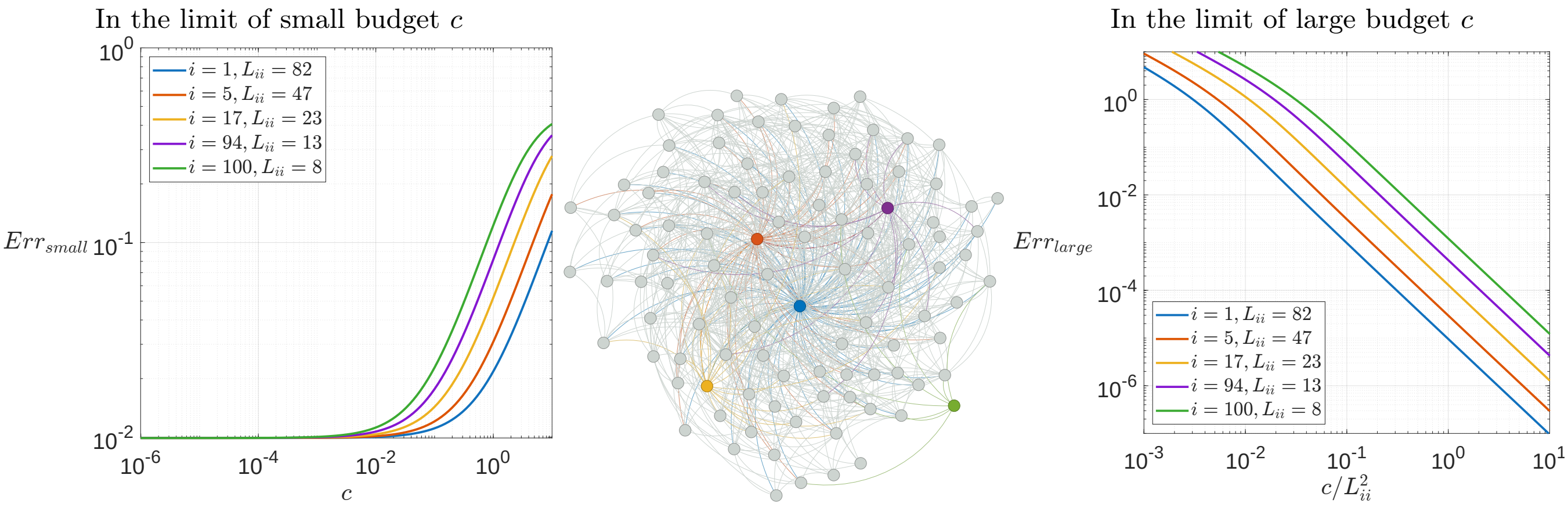

FIG. 3: The errors (Eq. (4)) of our scaling laws in the limit of small (left panel) and large (right panel) budget c. The indegree of node i is denoted by L_{ii}.

Furthermore, by using the formalism of the master stability function (MSF), changing the Laplacian matrix that is being used changes the values of the argument of the MSF that one needs to assess for stability but does not change the function itself. The different outcomes we see are related to a fundamental different behavior of the MSF for positive and negative values of its argument. See our response to Comment 2.1 above.

Comment Reviewer 2.4:

The characterization of the attack budget parameter c also requires more detailed analysis. The study refers to "small" and "large" budgets in the asymptotic analysis but does not provide quantitative definitions or specific threshold values that distinguish these regimes. It is also unclear how the budget relates to the natural coupling strengths in the network. For instance, are normal edge weights typically of order unity, and if so, what does it mean for the attack budget to be "small" or "large" relative to these values? The analysis would benefit from a discussion of how edge weight heterogeneity affects the conclusions and what constitutes a realistic attack budget in practical systems. These details are important for understanding the practical applicability of the theoretical results.

Answer to Reviewer 2.4:

We thank the Reviewer for their comment. In order to address this point raised by the Reviewer, we have performed a numerical study over our randomly generated scale-free network, Fig.3. We have seen that the following values of the budget can be considered ‘small’ and ‘large’ in our asymptotic analysis:

- the small budget should be $c < 10^{-2}$ (independent of the attacked node),
- the large budget should be $c > 0.1L_{ii}^2$ (dependent on the attacked node),

where L_{ii} is the indegree of the attacked node. The above values result in normalized prediction errors in order of 10^{-2}. The details of our analysis are available in the newly added Supplementary Note 9. We have commented on the above results in Remark 2 of the revised manuscript. For convenience, we have also copied the analysis below:

On the Magnitude of the Budget:

We comment on what values can be considered as ‘small’ or ‘large’ for the budget, so the proper scaling law is used. We perform a numerical study of the 100-node unweighted and undirected scale-free network in Fig. 3. We select 5 distinct network nodes to be attacked separately (bidirectional attack). For each attacked node, we vary the budget c, and evaluate the algebraic connectivity $f(L^b_{aug})$. Our goal is to see what values of the budget c can be considered as small or large, so that our scaling laws can be applied accordingly:

- For a small budget c: the algebraic connectivity $f(L^b_{aug}) = -c$, and
- For a large budget c: the algebraic connectivity $f(L^b_{aug}) = -2c + L_{ii}/2$.

where L_{ii} denotes the indegree of node i.

To assess the accuracy of the scaling laws above, we define the normalized errors corresponding to small and large values of the budget c, respectively, as:

$$Err_{small} = \left| \frac{f(L^b_{aug}) - (-c)}{f(L^b_{aug})} \right|, \tag{4a}$$

$$Err_{large} = \left| \frac{f(L^b_{aug}) - (-2c + L_{ii}/2)}{f(L^b_{aug})} \right|. \tag{4b}$$

We analyze the values of the two errors above to determine the orders in which the budget c can be considered small and large. Figure 3 shows the above errors for small and large values of the budget c. We choose the threshold 10^{-2} for both errors Err_{small} and Err_{large}. We see to have a low error of the predicted algebraic connectivity $f(L^b_{aug})$, the aforementioned bounds for small and large budgets can be considered.

Comment Reviewer 2.5:

The physical implementation of negative coupling also deserves more concrete discussion. The power grid example provides a clear illustration of how adversarial coupling can be realized through anti-phase oscillations. However, for the other systems mentioned, including consensus dynamics, formation control, and general synchronization problems, it remains unclear how adversarial negative couplings would actually be implemented in practice. What are the implementation constraints? Under what circumstances can an adversarial agent realistically establish such connections? A more thorough treatment of implementation feasibility across different application domains would strengthen the practical relevance of this work.

Answer to Reviewer 2.5:

We thank the Reviewer for this important comment. We agree that clarifying the physical implementation of negative (adversarial) coupling across different domains strengthens the practical relevance of our work.

In technological networks, we argue that the implementability of adversarial versus cooperative coupling reduces to a sign change in the feedback gain. In standard Laplacian coupling, the interaction takes the form $g(x_{\rm ref} - x_i)$, where $x_{\rm ref}$ is a reference state and x_i is the state of node i. Cooperative coupling corresponds to $g > 0$, while adversarial coupling corresponds to $g < 0$. Thus changing the sign of the feedback gain naturally yields either cooperative or adversarial coupling. For instance, in consensus or formation control systems, such a sign change can arise from faulty or malicious agents, incorrect controller design, or cyber-attacks that invert transmitted signals. Similarly, in power grid applications, adversarial coupling can be realized through devices enforcing anti-phase behavior, as discussed in Sec. IIC.

In biological systems, negative interactions are ubiquitous and naturally occurring. Examples include inhibitory synaptic coupling in neuronal networks, competitive interactions in ecological systems, and repression mechanisms in gene regulatory networks. In these contexts, "adversarial" coupling does not require external implementation, but is an intrinsic feature of the system dynamics. Our framework can thus be interpreted as capturing the effect of introducing or amplifying such inhibitory interactions in otherwise cooperative networks.

In social systems, negative coupling can arise through antagonistic or contrarian interactions. For example, in opinion dynamics models, individuals may adopt behaviors that systematically oppose those of their neighbors (so-called "contrarians"), effectively implementing a negative coupling. Similarly, misinformation or adversarial agents in online networks can act to disrupt consensus by promoting opposing states. These mechanisms provide realistic pathways through which adversarial interactions can be established.

Overall, while the specific implementation mechanisms differ across domains, negative couplings are both theoretically well-defined and practically realizable. In engineered systems, they can arise through faults or attacks that invert control signals; in biological and social systems, they are often intrinsic to the interaction structure. We will clarify these points in the revised manuscript.

We have better motivated the usage of negative interactions in the second paragraph of the Introduction and added a broad discussion on this point in the second paragraph of our Conclusion section.

Reviewer 3

Reviewer 3 evaluation:

Referee report for Paper (NCOMMS-26-012151): Extreme vulnerability to intruder attacks destabilizes network dynamics

In this paper, the authors study how a network can be destabilized by introducing a single adversarial node coupled via adversarial connections. They show the performance across several dynamical systems, including the synchronization of coupled chaotic oscillators, power grid dynamics, and phase synchronization based on Kuramoto dynamics. The main contribution of this work, as stated in the abstract, is that a single low-indegree node induces the greatest instability. The authors develop the theory for linear systems and demonstrate that it also extends to nonlinear systems. However, I have several critical concerns about the current version of the manuscript that prevent its publication.

Answer to Reviewer 3 evaluation:
We thank the Reviewer for their critical reading of our paper and for providing constructive criticisms.

Comment Reviewer 3.1:

The novelty of the manuscript remains unclear. There is a substantial body of literature on intruder attacks and adversarial perturbations in networks. The authors claim that their finding challenges the common assumption that hubs are the most critical nodes. However, I am not convinced that this result is particularly surprising, nor that it leads to a fundamentally new characterization of network vulnerability, as suggested. My understanding is as follows, and I would appreciate clarification if it is incorrect. For nodes with large indegree, the effect of an adversarial perturbation may be diluted due to the aggregation of many inputs. In this sense, it is not unexpected that low-indegree nodes could exhibit a stronger marginal impact. It may also be important to consider the role of outdegree, which reflects the extent to which a node influences others; nodes with higher outdegree may induce broader system-level effects. In addition, the authors show that the impact of a single node scales proportionally to 1/N. For linear systems, this behavior is not surprising and appears consistent with standard expectations.

Answer to Reviewer 3.1:
We thank the Reviewer for this thoughtful comment and for the opportunity to clarify the novelty of our work. We respectfully disagree that our findings can be explained solely by a simple dilution effect due to large indegree, or that they are a straightforward consequence of known linear scaling arguments.

First, while it is true that nodes with large indegree aggregate many inputs, our results go beyond this intuition. In particular, we provide a *spectral characterization* of how an adversarial perturbation affects the stability of the entire network, showing that the impact of a single intruder node is governed by the interplay between network topology and the non-normality of the dynamics. The identification of low-degree nodes as the most critical targets is not derived from a local input-averaging argument, but rather from their global effect on the algebraic connectivity and the associated transient growth of perturbations.

Second, while it is reasonable to expect that the outdegree of a node would play a role, since it reflects how strongly a node can influence the rest of the network, our analysis shows that this intuition does not hold in the present setting. In particular, our mathematical results demonstrate that the destabilizing effect of an adversarial node is governed solely by its indegree. This is not an assumption, but a direct consequence of our mathematical analysis involving the augmented Laplacian, which reveals that the relevant perturbation to the algebraic connectivity depends only on how the adversarial node receives inputs from the network, and not on how it distributes them. This result is therefore nontrivial and could not be inferred from heuristic degree-based arguments alone.

Third, regarding the observed $1/N$ scaling, we agree that similar scaling behaviors may arise, under specific conditions, in certain linear settings. However, our contribution is not limited to identifying this scaling, but rather to linking it to the rate of destabilization under adversarial perturbations and to the spectral properties of the augmented Laplacian. Importantly, we show that despite the $1/N$ decrease in average instability, a *single* adversarial node remains sufficient to destabilize the network irrespective of its size, which has significant implications for network robustness.

Overall, the novelty of our work lies in providing a unified framework that connects adversarial perturbations, graph properties, and transient dynamical effects, leading to a characterization of node criticality that differs from standard centrality-based or degree-based arguments. We have revised the manuscript to better emphasize these points and clarify the distinction from existing literature.

Comment Reviewer 3.2:

The connection between the theoretical developments in Sections A and B and the application section remains unclear to me, despite the authors' attempt to provide an explanation prior to Section A. The authors present f as a smooth function of c. However, the notion of "network destabilization" is not clearly defined. While several figures illustrate the system states over time before and after introducing the adversarial node, there is no quantitative measure of destabilization provided as a function of c. As a result, it is difficult to understand how the theoretical results relate to the observed dynamics. In particular, it is unclear whether destabilization should be interpreted as a continuous or discontinuous function of c, or how one would formally characterize this transition.

Answer to Reviewer 3.2:
We thank the Reviewer for this insightful comment and for the opportunity to clarify the connection between the theoretical developments and the application results. In the revised manuscript, we have rewritten the text following Eq. (6) to explicitly define what we mean by a *destabilization.* All of our changes are highlighted in blue on page 3 of the paper. We copy below the most important changes:

Consensus is achieved if $\|\delta\mathbf{X}(t)\| \to 0$ *as* $t \to \infty$. *In our setting, the original Laplacian matrix L is proper, which guarantees that consensus is achieved prior to the attack. However, the addition of a single adversarial connection with the intruder implies that the resulting augmented Laplacian matrices* L^b_{aug} *and* L^u_{aug} *are no longer proper, as they are known to possess at least one eigenvalue with negative real part (see e.g., [16,17]) and, as a consequence, consensus is not achieved. We refer to this transition from a stable regime (before the adversarial connection) to an unstable one (after its introduction) as a* destabilization.

The Reviewer is correct that we present f as a smooth function of c, see in particular our Eq. (8) and Fig. 3. However, $\|\delta\boldsymbol{X}(t)\|$ in Eq. (7), which defines a destabilization, is not a continuous function of c and will change abruptly when one transitions from a stable system to an unstable system, see for example our Figs. 5,6, and 7. We have rewritten the text on Page 3 to make this clear and checked carefully for our usage of the word 'destabilization' throughout the manuscript to make sure it is used consistently.

In this paper, we focus on the algebraic connectivity f, which determines the transient exponential growth rate of the norm of the perturbation immediately after the attack. This definition provides a direct quantitative link between the theory and the simulations: the time evolution of the system states shown in the previously mentione dfigures reflects the sign and magnitude of f. Larger values of $-f$ correspond to faster divergence from the synchronized state, as observed in the numerical results.

We also remark that the figures comparing system behavior before and after the attack involve two different systems: an N-dimensional system (without the intruder) and an $(N+1)$-dimensional system (with the intruder).

Comment Reviewer 3.3:

The writing of the manuscript requires substantial improvement. References are not presented in proper order, and the exposition is often difficult to follow. Given that the journal targets a general audience, key concepts should be clearly defined both in text and, where appropriate, mathematically. Terms such as instability, destabilization, and most valuable components of a network are introduced without precise definitions, which makes it difficult to interpret the results.
In Equations (4) and (5), the authors introduce the variables b and c, but their roles are not immediately clear. In particular, it is not evident how one should choose b under a given budget constraint. Should the values b_i follow a specific distribution? Only later does it become apparent that b_i=-1 (or other value) when the new node connects to node i, and b_i=0

otherwise. More broadly, the logical flow is sometimes difficult to follow. For example, in the second paragraph of the introduction, the authors first distinguish between cascading failures and instability, but then immediately state that the paper focuses on the effects of adversarial connections. The relationship between these concepts is not clearly articulated, leaving the reader uncertain about how the discussion of instability connects to the study of adversarial connections.

Answer to Reviewer 3.3:

We are very much indebted to the Reviewer for their feedback. We have done our best in rewriting the paper and improve readability. These changes are discussed next.

- We have used the proper style file for the bibliography of papers published in Nature Communications.

- We have added explanations for the physical meaning of the quantities b and c, right after Eqs. (4) and (5). Given c, the particular choice of the b's is dictated by the particular solutions of of the optimization problems introduced in Problems 1 and 2.

- We have added rigorous mathematical definition for terms such as instability and destabilization. We did our best at introducing the several components of a network that are used in our analysis.

-We also agree with the Reviewer that the second paragraph from the Introduction was unclear. It has now been completely rewritten and broken into two separate paragraphs as follows:

"Alternatively, network instabilities may arise from the presence of adversarial connections between the nodes of a network [15-17]. In contrast to cascading failures, which are triggered by the removal of nodes or edges, adversarial interactions correspond to the addition of links with negative weights. Such interactions have been reported in brain networks as inhibitory connections between neurons [17], in social networks as antagonistic relationships between agents [15], and in distributed control systems as faulty or corrupted communication processes [16].

While both cascading failures [9,16-20] and instabilities induced by adversarial interactions can disrupt network functionality, they operate through fundamentally different mechanisms. Cascading failures typically unfold as discrete, far-from-equilibrium processes, whereas adversarial interactions destabilize an existing equilibrium through continuous dynamical effects. In this work, we focus on the latter mechanism, namely the destabilization induced by adversarial connections. Although distinct in nature, both edge removals and adversarial interactions can ultimately lead to network instability."

Comment Reviewer 3.4:

The assumptions of the manuscript appear either unrealistic or insufficiently general. In the abstract, the authors state that they address the question of how "one or a few intruders" affect network stability; however, the analysis in the paper focuses exclusively on a single-node perturbation. It would be important to understand how the results extend to cases where a fraction of nodes is perturbed. The authors further claim that a single low-indegree node induces the greatest instability. This claim would benefit from more extensive validation, for example through simulations across a range of dynamical systems, parameter settings, and network structures. In Equations (4) and (5), the authors consider two extreme cases—bidirectional and unidirectional connections. It would be more informative to examine intermediate or more general configurations, such as a node that points to s nodes and is influenced by r nodes.

Answer to Reviewer 3.4:

We very much appreciate this comment. In the abstract, we replaced "one or a few intruders" with "a single intruder", to be more precise.

We have addressed the case of multiple attacks in the newly added Supplementary Note 2 in which we discuss the case of multiple attacks. In summary, we see that still the optimal attack, one that minimizes the algebraic connectivity of the network, is the attack with all budget concentrated in one link from one of the intruders toward the node with the lowest indegree, and all other intruders remain disconnected. There is no difference on which intruder is chosen as the attacker, since all are identical before the attack. These empirical results are summarized in Remark 1 (on page 10) of the revised manuscript. Below are the details of our analysis, copied from the newly added Supplementary Note 2, where we discuss the case of an attack involving multiple intruders.

We start by distinguishing between the two cases of

1. multiple attacks one-by-one,
2. multiple attacks simultaneously.

In case 1, intruders attack the network sequentially. For this case, our analysis and results already stated in the main manuscript hold, i.e., for each new attack, the intruders should target the node with the lowest indegree. The reason is that the optimal first attacks will be on the node with the lowest indegree. After the first attack, the indegree of the attacked node will be reduced by the budget amount, while the indegrees of all other nodes remain intact. As a result, when the second attack is about to happen, the attacked node still has the lowest indegree, and thus, is the optimal candidate for the second attack as well. By induction, all one-by-one attacks should target the node with the lowest indegree for maximum effect of inducing transient instability.

For case 2, we form an optimization for the simultaneous attacks, inspired by our optimization for a single attack. For simplicity, we consider $m \geq 1$ simultaneous bidirectional attacks on an undirected network with N nodes. Now, the links between the intruders and the original network can be characterized by the matrix $B \in \mathbb{R}^{N\times m}$, where entry $B_{ij} \leq 0$ denotes the strength of the attack from intruder j to node i. Similar to the case of one attack, we set the prescribed budget of the attack to be $-c$, with $c \geq 0$, and require the sum over all the attack links (between the intruders and the network nodes) to be the budget, i.e., $\sum_{i=1}^{N}\sum_{j=1}^{m} B_{ij} = -c$. We aim to find the optimal attack strategy, i.e., the optimal matrix B such that the algebraic connectivity of the following augmented Laplacian matrix is minimized:

$$L_{aug}^{b} = \begin{bmatrix} L + \text{diag}(B\mathbf{1}_m) & -B \\ -B^{\top} & \text{diag}(B^{\top}\mathbf{1}_N) \end{bmatrix}. \tag{5}$$

Here, $\mathbf{1}_N$ denotes a vector of length N with all entries equal to 1.

Given the symmetric N-dimensional Laplacian matrix L, budget $c > 0$, and the $N \times (N+1)$ matrix $V \in \text{Null}(\mathbf{1}_{N+1}^{\top})$ with orthonormal columns, we solve for the optimal attack connections $B \in \mathbb{R}^{N\times m}$:

$$f(L_{aug}^{b}) = \min_{B,\boldsymbol{x}} \quad \boldsymbol{x}^{\top}\left(V^{\top}L_{aug}^{b}V\right)\boldsymbol{x} \tag{6a}$$

$$\text{s. t.} \quad \boldsymbol{x}^{\top}\boldsymbol{x} = 1, \tag{6b}$$

$$L_{aug}^{b} = \begin{bmatrix} L + \text{diag}(B\mathbf{1}_m) & -B \\ -B^{\top} & \text{diag}(B^{\top}\mathbf{1}_N) \end{bmatrix}, \tag{6c}$$

$$B_{ij} \leq 0, \quad i = 1, \dots, N, j = 1, \dots, m, \tag{6d}$$

$$\sum_{i=1}^{N}\sum_{j=1}^{m} B_{ij} = -c. \tag{6e}$$

The global solution of the above optimization gives the optimal m simultaneous attack strategy, $B^{\star}$, that minimizes the algebraic connectivity $f(L_{aug}^{b}) < 0$, which results in the highest rate of transient instability (minimizing the algebraic connectivity).

We performed extensive numerical simulations where we generated random networks (randomly generated Laplacian matrix L), chose a random budget amount c and random number of attackers m, and solved the optimization in Eqs. (6). Our empirical results show that the optimal attack is to concentrate all the budget $-c$ in one undirected link between any of the intruders and the node with the lowest indegree, i.e., $B^{\star} = [B_{ij}^{\star}]$ such that

$$\begin{aligned} &B_{kl}^{\star} = -c, \quad k = \arg\min_{i} L_{ii}, \quad \text{and } l \text{ is chosen arbitrarily from } 1, \dots, m, \\ &B_{ij}^{\star} = 0, \quad \forall i \neq k, \forall j \neq l. \end{aligned} \tag{7}$$

This suggests only one intruder, say intruder l, actually should attack the network with all the budget concentrated in one link toward the node with the lowest indegree, and all others remain disconnected. There is no difference on which intruder is chosen as the attacker (as intruder l), since all intruders are identical before the attack. This finding strengthens our results in the case of one intruder attack by further emphasizing that the optimal attack is to target the node with the lowest indegree and concentrate all budget on one link. We leave the formal proof for future work and conclude this section with an example.

We take the undirected 7-node network from the power grid example in the main manuscript. Given a budget $c = 1$, what is the optimal bidirectional attack on the network, given m intruders? Our numerical results suggest that the optimal attack is to concentrate all the budget in one undirected connection between any of the intruders and the node with the lowest indegree. Figure 1 demonstrates these results by showing that the optimal attack in the case of $m = 1, 2, 3$ intruders is to have only one connection, with all budget concentrated in it. For all cases with $m = 1, 2, 3$, the resulting algebraic connectivity after the optimal attack is $f(L_{aug}^{b}) = -1.7058$. This indicates the minimum algebraic connectivity $f(L_{aug}^{b})$ is not a function of the number of intruders m, but a function of the total

budget c, which is kept the same for all m. Therefore, the empirical results in Eq. (7) are confirmed through this example.

Regarding the attack on a portion of nodes, we comment that we do not 'force' the attack on one node. We aim to find the most disruptive attack given a budget (the weight of the connection between the intruder and the network nodes). To find the most disruptive attack, we form and solve an optimization problem in which the algebraic connectivity of the network after the attack is minimized, which results in the highest rate of transient instability. Our result is that the most effective attack is to target the node with the lowest indegree.

In this work, we have applied this result in three different applications: Synchronization of coupled chaotic oscillators, the power grid, and formation control. Since our goal is to report the most effective attack, and also since we obtained that, regardless of the application and the dynamical system setup, the most disruptive attack is to target the node with the lowest indegree, we have left the study of further applications to future studies.

Finally, we remark that the point of this paper is to study the topological configuration of a network after an attack that would result in the largest transient instability, which is the reason why we have focused on the two relevant cases of a unidirectional connection and a bidirectional connection between the intruder and the attacked node(s). While other possible cases, such as those that are mentioned by the Reviewer, are interesting, we find that these additional problems are substantially more complex and would require a separate study. We thank the Reviewer for pointing this out.

Comment Reviewer 3.5:

There also appears to be an inconsistency in the mathematical treatment. In Equation (7), the norm of the perturbation is computed by summing over all N+1 nodes, whereas in Equations (13) and (18), the summation appears to involve only N nodes. This discrepancy should be clarified.

Answer to Reviewer 3.5:

We thank the Reviewer for noting this. In the revised manuscript and revised Supplementary Information, all perturbation evaluations are performed over N nodes. We have made sure our formulation is consistent.

Comment Reviewer 3.6:

The figure 1 do not clearly convey the main ideas of the paper. As currently presented, they are difficult to interpret and do not provide sufficient support for the claims being made.

Answer to Reviewer 3.6:

We thank the Reviewer for this helpful comment. We agree that the previous version of Fig. 1 did not sufficiently convey the main ideas of the paper and could be difficult to interpret. For this reason, the figure has been redesigned. The revised figure now presents a clearer comparison between the unperturbed case (no attack) and the perturbed case (presence of an intruder connected through an adversarial link).

We emphasize that the purpose of Fig. 1 is to illustrate the general mechanism of an intruder attack. In the revised version, the attack is shown targeting a low-indegree node, in order to remain consistent with the analytical results developed later in the manuscript, where such a strategy is shown to maximize vulnerability.

Finally, we stress that Fig. 1 is intended as a schematic illustration rather than as quantitative support for the claims. We have revised the labels and caption accordingly to guide the reader more effectively and to ensure full consistency with the rest of the paper.

Reviewer 1

Reviewer 1 evaluation:

The authors have addressed some of my concerns and, from their reply, I better understand the main message of their work. Still, I think that the new version of the manuscript hasn't improved as much and I wish that the clarity of their reply could reflect in the manuscript as well. I still think that this work is relevant and the main result is interesting. I believe that, with some effort in the revision of the manuscript, it can be ready for this journal.

I only have a few comments:

Answer to Reviewer 1 evaluation:
We thank Reviewer 1 for their positive assessment of our work.

Comment Reviewer 1.1:

Because I now understand much better some points (thanks to the reply), I have an additional question. what happens if the node with lowest degree has only one link to the hub? For example, in Fig 5A, if the green node is connected only to the blue node (hub), would an attach on the green node effective or would the hub somehow absorb the attack? I think such points need to be addressed, because the claims of the authors are very strong and general.

Answer to Reviewer 1.1:
We thank the Reviewer for this insightful comment. To illustrate the robustness of our result, we randomly generate an 18-node undirected network (left panel in Fig. 1 below), for which node 1 has the highest degree ($\deg_1 = 10$), which we call the 'hub node'. Nodes 17 and 18 have the lowest degree ($\deg_{17} = \deg_{18} = 1$), which we call the 'leaf nodes'. The difference between the two leaf nodes is that node 18 directly connects to the hub, but node 17 does not. We perform the same numerical exercise that we present in the manuscript for the case of synchronization, where Lorenz oscillators (with the same local function $\boldsymbol{F}$, and coupling function $\boldsymbol{H}$ as in the main manuscript) are connected through this 18-node network. We set the coupling strength $\sigma = 9$, and the budget for the attack to be $c = 1$. We see from Fig. 1 (right panel) that the rate of transverse instability of the synchronization dynamics is the same when nodes 17 and 18 are attacked. The algebraic connectivity after the attack is $f = -1.6$ for both cases when nodes 17 and 18 are attacked. This shows that the vulnerability to intruder attacks is only affected by the indegree of the attacked node, not its neighbors, since the change in the algebraic connectivity is only a function of the node degree (or indegree for directed graphs) as extensively discussed in the main manuscript. This study has now been included in the new Supplementary Note 9.

Comment Reviewer 1.2:

On the same line, I think the issue of the attraction basin needs to be tackled, at least from some angle, and I disagree with the authors that it goes beyond the scope of this work. Again, given the strong claims, it is important to know whether the system can go back to the equilibrium and how quickly once the attack has stopped.

Answer to Reviewer 1.2:
We thank the Reviewer for this insightful comment. The study of basins of attraction is notoriously difficult and is typically carried out numerically on a case-by-case basis. Consequently, determining whether stability is recovered after an attack ends generally requires detailed knowledge of the underlying nonlinear dynamics. As a result, any conclusions are often problem-specific and, in many cases, parameter-dependent, limiting both their generality and broader applicability. Having stated this, we will try to respond at the best of our capabilities. Here, we try to state a general methodology in which the transient rate of stability/instability may be utilized to conservatively approximate the basin of attraction. The basin of attraction can be calculated/approximated by studying the asymptotic stability of the trajectories initiated from various points in space.

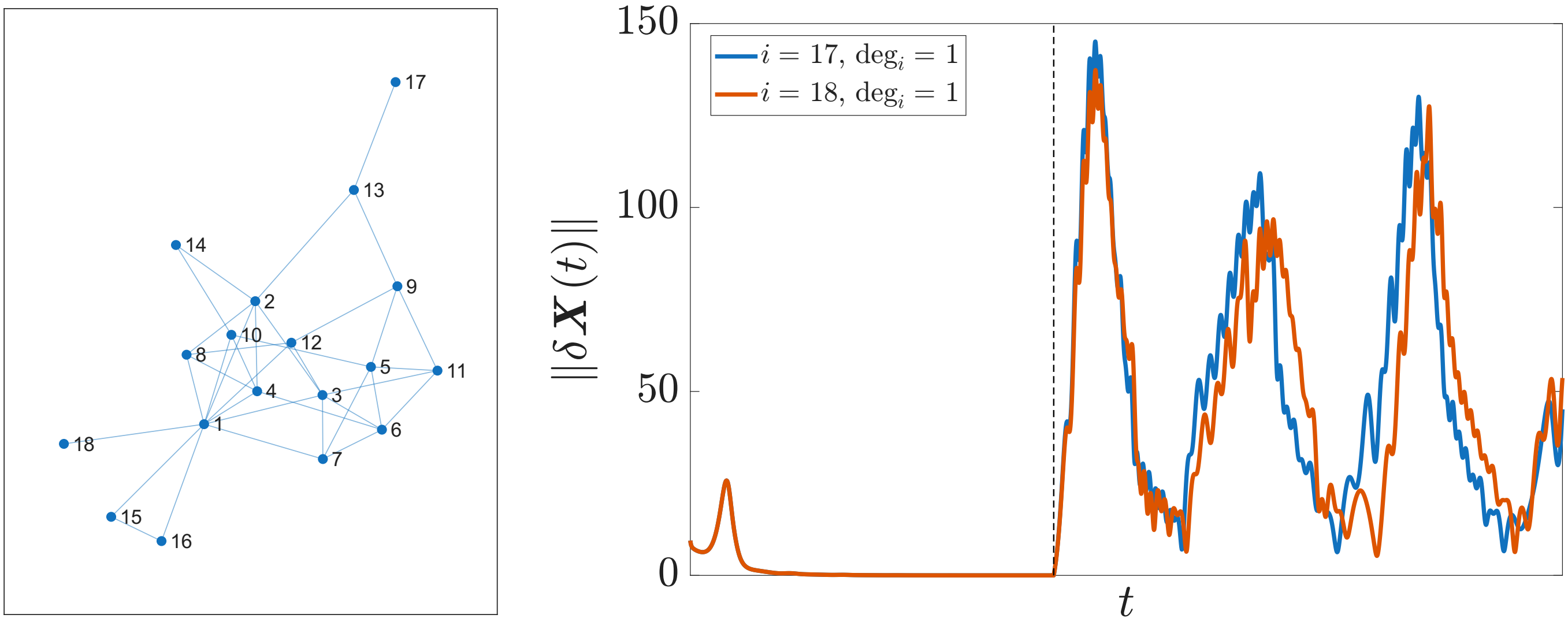

FIG. 1: The left panel shows a randomly generated unweighted and undirected network. The right panel shows the norm of the transverse perturbations $\|\delta \boldsymbol{X}(t)\|$ when either one of the leaf nodes 17 and 18 is attacked.

A recent preprint [1] connects the notion of the transient rate of instability/stability, which is referred to as 'reactivity', to the asymptotic rate of growth/decay, which here is MLE. Similar to the MLE, if the trajectory is transiently divergent, the reactivity is positive, and if it is transiently converging, the reactivity is negative. This work creates the connection by showing, in the context of discrete-time systems or generally 'maps', that the reactivity of the p times iterated map converges to the MLE in the limit of $p \to \infty$. Now, one may aim to approximate the basin of attraction by evaluating the reactivity of the dynamics in various points in space, and conclude that the basin must include the points that have a negative reactivity. This connection is explicitly and rigorously established by contraction theory (see e.g., Lohmiller, W., Slotine, J.-J. E. (1998). On contraction analysis for nonlinear systems. Automatica, 34(6), 683–696.)

In our paper, we focus on the transient rate of instability, which is the special case of positive reactivity induced by an intruder attack. The definition of the algebraic connectivity of the Laplacian matrix is exactly the negative of the transverse reactivity of the consensus dynamics. In a single-integrator consensus dynamics, the basin of the consensus manifold is the entire phase space (with the only exception of the consensus manifold.) After the attack, we show that the algebraic connectivity is negative, thus the reactivity is positive. Since in our Theorem 1 (in Methods A), we prove that the reactivity is greater than or equal to the spectral abscissa, it follows that the spectral abscissa is also positive, leading to asymptotic instability. Once the attack is concluded, the algebraic connectivity becomes positive, thus the reactivity becomes negative and so does the spectral abscissa. Therefore, the dynamics becomes globally transversely stable.

In other applications, such as synchronization, power grid dynamics, and Kuramoto oscillators, it is difficult to comment on the basin of attraction as we do in the case of single-integrator consensus dynamics. These applications can be highly nonlinear, and each case requires a specific study that reflects the particular form of the equations and of the nonlinearity involved. Consequently, the conclusions may differ substantially from one particular choice of the equations to another.

---

[1] Nazerian, A., Sorrentino, F. & Aminzare, Z. Bridging the gap between reactivity, contraction, and finite-time lyapunov exponents (2025). URL `https://arxiv.org/abs/2410.23435`. 2410.23435.

Comment Reviewer 1.3:

Minor comments:

_in the abstract the authors wrote "an intruder agents"

_page 2, in the part in blue in the left column: need not "to" spread?

Answer to Reviewer 1.3:
We thank the Reviewer for pointing this out. We have fixed both problems.

Reviewer 2

Reviewer 2 evaluation:

The authors have responded to my previous comments in a thorough and largely satisfactory manner. I have two remaining minor points for the authors.

Answer to Reviewer 2 evaluation:
We thank Reviewer 2 for reading their careful assessment of the paper.

Comment Reviewer 2.1:

Regarding Comments 2.1 and 2.2:
The explanation that adversarial coupling shifts the Laplacian spectrum toward negative values, requiring evaluation of the Master Stability Function at negative arguments, is convincing, and the MSF plots in Supplementary Note 6 provide useful supporting evidence. However, the logical chain connecting these observations to the conclusion that targeting low-indegree nodes produces greater instability is currently spread across the main text and supplementary materials. I recommend adding a concise summary of this reasoning in the main text to improve readability.

Answer to Reviewer 2.1:
We thank the Reviewer for pointing this out. Following the Reviewer's suggestion, we have expanded the discussion of our results in the context of the Master Stability Function framework. Specifically, we have added an extended discussion on Page 7 (highlighted in blue in the revised manuscript) that clarifies the connection between our approach and the Master Stability Function formalism. This new material reflects and elaborates on the arguments presented in our original response to the Reviewer.

Comment Reviewer 2.2:

Regarding Comment 2.3:
The authors' theoretical position is internally consistent: the main result, namely that attacking the node with the smallest L_{ii} is optimal, holds for a general weighted Laplacian. However, the interpretation that low-indegree nodes are the most vulnerable is contingent on the specific Laplacian formulation adopted. Under a normalized Laplacian, where all diagonal entries are equal, this degree-dependent vulnerability disappears. The manuscript currently presents the vulnerability of low-indegree nodes as a broadly applicable finding throughout the abstract, introduction, and conclusions, without acknowledging this dependence. I recommend that the authors moderate the tone of this claim accordingly and clarify that the degree-dependent interpretation arises specifically from the modeling choices embedded in Eq. (1).

Answer to Reviewer 2.2:
We thank the Reviewer for their comment. We just note here that the paper provides the following definitions of indegree and outdegree of a node: *The network connectivity is described by a digraph with adjacency matrix $A = [A_{ij}]$, where A_{ij} is the strength of the directed coupling from node j to node $i \neq j$, $A_{ij} > 0$ indicates a cooperative interaction (also sometimes called mutualistic or attractive), $A_{ij} < 0$ indicates an adversarial interaction (also sometimes called competitive or repulsive), and $A_{ij} = 0$ indicates no interaction.* The indegree of node i is equal to $d_i^{in} = \sum_{j \neq i} A_{ij}$ and the outdegree of node i is equal to $d_i^{out} = \sum_{j \neq i} A_{ji}$. We note that the adoption of the graph Laplacian is justified because it naturally describes a broad class of diffusively coupled systems and constitutes one of the standard coupling operators in network dynamics, arising in numerous physical, biological, and engineering applications, including electrical (gap-junction) coupling in neuroscience.

That said, we thank the Reviewer for this insightful observation. We agree that if the coupling weights are normalized so that each node has the same total coupling strength, then the diagonal entries become identical and, at

the level of the algebraic connectivity, the node to which the adversarial node is attached is no longer distinguished. In fact, our large-cost asymptotic result (Secs. IV.F,G) depends on the diagonal entry L_{ii} of the adopted coupling operator. Consequently, for normalized Laplacians such as the random-walk Laplacian, whose diagonal entries are identical (in our sign convention, $L_{ii} = -1$), the criterion based on L_{ii} implies that the effects of attacking different nodes are the same. We have added a remark on Pag. 5 of the main manuscript to clarify that the node-level interpretation is specific to the choice of coupling operator.

To conclude, in order to address this comment by the Reviewer we have: (i) added text to the abstract, introduction and conclusion to exactly clarify our definition of indegree and (ii) added a remark on Pag. 5 clarifying that the interpretation of low-indegree vulnerability follows from the specific Laplacian definition through its diagonal entries L_{ii}. See parts of the revised manuscript highlighted in blue.

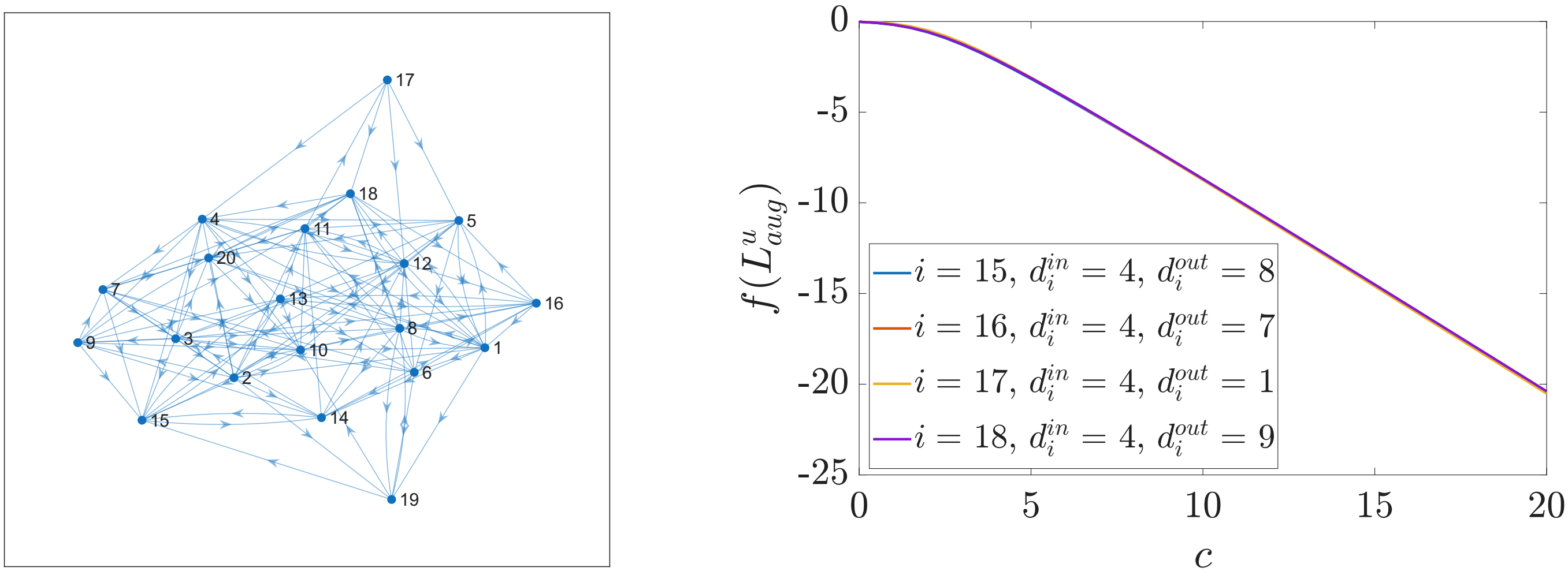

FIG. 2: Left panel shows the randomly generated directed and unweighted Erdos-Renyi graph. The right panel shows the algebraic connectivity $f(L^u_{aug})$ after a unidirectional attack on node i, where $i = 15, 16, 17, 18$, while the budget c is varied.

Reviewer 3

Reviewer 3 evaluation:

Referee report for Paper (NCOMMS-26-012151A): Extreme vulnerability to intruder attacks destabilizes network dynamics

I appreciate the authors' efforts to revise the manuscript and address the comments from both the other reviewers and me. However, I have several additional comments regarding the revised manuscript and the response letter.

Answer to Reviewer 3 evaluation:
We thank the Reviewer for their critical reading of our paper and for providing constructive criticisms.

Comment Reviewer 3.1:

I understand your spectral analysis about the Laplacian and obtaining the critical low-degree nodes from optimization of the global effect but local analysis. As you know, many studies showed the connections between the network eigenvalue properties and degree/degree distribution. (for example Allesina, Stefano, and Si Tang. "Stability criteria for complex ecosystems." Nature 483.7388 (2012): 205-208.) Therefore, it reasonable to add some controlled experiments about the effect of in- and out- degree on its destabilization.

Answer to Reviewer 3.1:
We thank the Reviewer for this thoughtful comment and for the opportunity to clarify the novelty of our work. Following the Reviewer's input, we have performed a numerical study in which we generate a random directed and unweighted Erdős-Rényi graph (shown in Fig. 2 below left panel). We set up attacks on nodes 15, 16, 17, and 18 that have the same in-degree $d_i^{in} = 4$, but different out-degrees. We sweep over the budget c from 0 to 20 and evaluate the algebraic connectivity of the unidirectional attack $f(L^u_{aug})$. The results are shown in Fig. 2 right panel. We see that although the nodes have different outdegrees, the result of the attack on nodes with the same indegree is the same, since the resulting algebraic connectivity after the attack is the same. A reference to the paper mentioned by the Reviewer has been added to our conclusions.

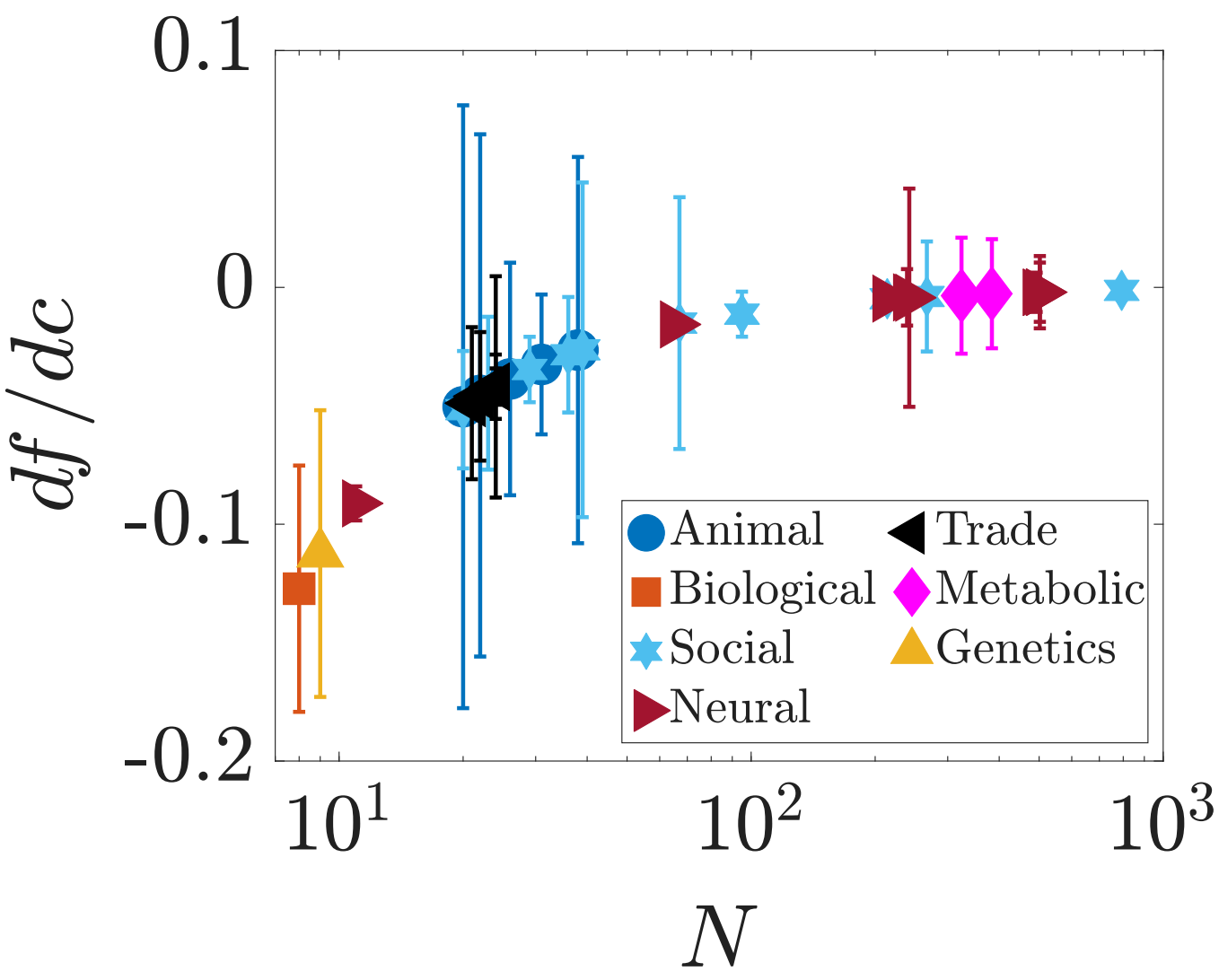

FIG. 3: The mean slope df/dc along with its standard deviation (as vertical bars) for unidirectional connections of the adversarial agent for selected real networks with size N.

Comment Reviewer 3.2:

The statement that there is a "(1/N) decrease in average instability" would be more convincing if accompanied by a characterization of the underlying statistical distribution. In particular, the authors should report the variance or dispersion of node-level effects, since the mean may mask substantial heterogeneity. Why do some low-degree or high-degree nodes exert very different impacts on instability?

Answer to Reviewer 3.2:
We thank the Reviewer for this insightful comment. To address this point, we have computed the mean and standard deviation of the slope for unidirectional attacks using data from real networks. The results are shown in Fig. 3. We see that, unlike the mean, the standard deviation is not correlated with the network size N. This figure was included in the Supplementary Note 4.

Comment Reviewer 3.3:

In the revised version, the authors define destabilization as a transition from a stable regime to an unstable one. However, the manuscript does not clearly specify what constitutes a stable versus an unstable regime. In control theory and dynamical systems, stability is usually defined relative to an equilibrium point. For a linear system, or a nonlinear system linearized around an equilibrium, local stability requires the eigenvalues of the system matrix or Jacobian to have negative real parts. Therefore, destabilizing means the local stability condition does not satisfy. What you intended to define is more about "desynchronization" or other related terms.

Answer to Reviewer 3.3:
We thank the Reviewer for giving us an opportunity to further clarify this point. Our definition of destabilization, which appears after Eq. (7) is provided in the context of linear consensus dynamics. It is very similar to the definition of stability of a fixed point of a linear dynamical system, with the caveat that we study stability of the consensus manifold and not a single fixed point. Because the Laplacian matrix has always at least one zero eigenvalue and the corresponding right eigenvector is $[1, 1, ..., 1]$, by construction the motion along the consensus manifold is marginally stable. Accordingly, when we talk about stability we mean 'transverse stability'. The system is stable if all the other eigenvalues of the Laplacian are positive (would be negative, but note the negative sign in front of the Laplacian matrix in Eq. (6)) and is unstable if at least one of the other eigenvalues is negative. More specifically, the addition of

the intruder has exactly the effect of transforming the Laplacian matrix from one with one zero eigenvalue and other eigenvalues positive to one with at least one negative eigenvalue. Following the Reviewer's input (see also Comment 3.4), we have introduced a formal definition of destabilization, see the new Definition 1, right after Eq. (7).
In Section C 'Applications' we present other cases of dynamics which we investigate from the point of view of intruder attacks: synchronization, power grid dynamics, distributed formation control. That section starts with this statement: 'Although our results are derived for the case of the consensus dynamics (6), they have direct implications in the case of several real-world applications, described by the general set of equations (1). Below we discuss applications of interest in the realm of man-made networks.' Following the Reviewer's imput, we have now added Sections I, J, and K of the Methods, where we present a detailed asymptotic stability analysis for the cases of Synchronization Dynamics (Sec. I), Power Grid Dynamics (Sec. J) and Formation Control Dynamics (Sec. K.) Similarly, we have also added Methods Section L where we present the stability analysis for the case of the Kuramoto dynamics.

Comment Reviewer 3.4:

A further concern with the current definition is its treatment of partial synchronization. The authors define the order parameter in Eq. (19), where ($\rho = 1$) corresponds to complete synchronization and ($\rho \approx 0$) corresponds to a random or incoherent state. Yet, depending on the system parameters and network topology, the system may settle into partially synchronized states with intermediate values of (ρ), such as 0.9, 0.7, or lower. Under the authors' framework, how should the stability of such partially synchronized states be quantified? These ambiguities suggest that the manuscript needs a formal mathematical definition of destabilization, one that is applicable at least to the specific dynamical systems analyzed in the paper. It is better to be applicable to other similar dynamics not studied here.

Answer to Reviewer 3.4:
We first emphasize that our general assumption throughout the manuscript is that the underlying system has a well defined state, which is locally stable before introduction of the intruder and becomes unstable after introduction of the intruder. To address the comment by the Reviewer, in the revised manuscript we have made the following two changes: (i) We have introduced a formal definition of stability for the case of the consensus dynamics, see the new Definition 1 and (ii) we have now added rigorous theoretical derivations that explain how the well behaved state can be destabilized in the cases of Synchronization Dynamics (Methods Sec. I), Power Grid Dynamics (Methods Sec. J), Formation Control Dynamics (Methods Sec. K), and Kuramoto Dynamics (Methods Sec. L.) For example, in the case of synchronization, we see that instability (loss of synchronization) can be achieved with the introduction of a single intruder connected with a single adversarial connection to the rest of the network, provided that the associated master stability function is positive for negative values of its argument. This appears to be always satisfied in all our computations of the master stability function, see Methods Sec. K. The Reviewer's question focuses on the interesting case of the Kuramoto dynamics. We appreciate the comment and the opportunity to respond. As mentioned, our analysis applies to a state that is stable before introduction of the intruder; such a state may be one that is perfectly synchronized ($\rho = 1$) or close to synchronization ($\rho < 1$). A sufficient condition for this state to be stable is that the Laplacian matrix L' (see Methods Sec. L) is proper prior to introduction of the intruder. We also show that a sufficient condition for this state to become unstable is that the Laplacian ceases to be proper after introduction of the intruder. Specifically, a straightforward consequence of Theorem 1 in the Methods is exactly that the introduction of a single intruder connected with a single adversarial connection to the rest of the network is sufficient to make the Laplacian non-proper and thus to destabilize the dynamics. To conclude, our result is not limited to a specific ρ, but just to a case for which the Laplacian matrix L' is proper prior to the attack. We have added a comment to make this clear.

Editor

Editor evaluation:

Thank you for submitting your manuscript “Extreme vulnerability to intruder attacks destabilizes network dynamics" to Nature Communications. I am delighted to say that we are happy, in principle, to publish it under an open access license.

First, we ask you to revise your paper to address our editorial requests (in the attached Author Checklist) and any remaining comments from reviewers (included at the end of this email, if applicable).

Failure to comply with our editorial requests will result in further revisions and delays in accepting your manuscript. Please also see the Nature Communications formatting instructions for further information.

We look forward to seeing your revised manuscript. Please let us know if the process may take longer than two weeks.

Answer to Editor evaluation:
We would like to thank the Editor for their timely handling of our paper NCOMMS-26-012151B, entitled “Extreme vulnerability to intruder attacks destabilizes network dynamics." We have addressed all comments by the three Reviewers, as explained in the detailed responses that follow. All changes in the main draft are highlighted in blue.

Sincerely,

The Authors

Reviewer 1

Reviewer 1 evaluation:

The authors satisfactorily addressed all my concerns. I think this paper is ready to be be published in this journal.

I only have very minor comments.

Answer to Reviewer 1 evaluation:
We thank the Reviewer for their critical reading of our paper and for providing constructive criticisms.

Comment Reviewer 1.1:

at the beginning of Sec I (page 17, first column) one reference didn't pass and there is a "?"

Answer to Reviewer 1.1:
We thank the Reviewer for mentioning this issue. We have fixed the reference.

Comment Reviewer 1.2:

Fig. 10 in the Supplementary is very interesting. I think the authors could add a few more figures with larger networks to show that this works even when the degree of the hub is much higher (given that real networks can have hubs with degrees of also the order of hundreds). This would further strengthen their claim.

Answer to Reviewer 1.2:
We thank the Reviewer for this thoughtful comment. We have included a new figure in the SI, Supplementary Fig. 11, that exactly shows what the reviewer requested. This figure shows a scale-free network with 200 node and a hub with degree 195. When leaf nodes are attacked, while one is connected to the hub and the other is not, the rate of the transient instability of both attacks is the same; thus, once again confirms our theoretical results in the main paper that the vulnerability to intruder attacks are solely a function of (in)degree of the attacked nodes.

Reviewer 2

Reviewer 2 evaluation:

The authors carefully revised the ms according to the reviewers' comments. I believe that the current version of the ms meets the criteria for publication.

Answer to Reviewer 2 evaluation:
We thank the Reviewer for their critical reading of our paper and for providing constructive criticisms.

Reviewer 3

Reviewer 3 evaluation:

Referee report for Paper (NCOMMS-26-012151B): Extreme vulnerability to intruder attacks destabilizes network dynamics

The authors have revised the manuscript and addressed most of my previous comments. Therefore, I only have the last remaining major comment regarding the new results about the detailed analysis of -1/N.

Answer to Reviewer 3 evaluation:
We thank the Reviewer for their critical reading of our paper and for providing constructive criticisms.

Comment Reviewer 3.1:

According to Equ. (10) and supplementary fig 5, for unidirectional, $< df/dc >= 1/N$ because df_i/dc is close to -1 for all L_{ii}, which is independent of network sizes (at least for $N = 31$ and 26). However, for the bidirectional case, df_i/dc is close to 0 for each L_{ii}. Therefore, the sum of df_i/dc is about $-N$ for the unidirectional case but 0 for the bidirectional case. This is why $< df/dc >$ is not a smooth function of N for the bidirectional case. Cearly, there is some consistent behavior, and some normalization may make them comparable.

Answer to Reviewer 3.1:
We thank the reviewer for this insightful observation and for suggesting that an appropriate normalization may reveal a common scaling behavior for the two types of attacks.

We first clarify that the unidirectional and bidirectional cases appear to have been interchanged in the comment. In Supplementary Fig. 5, the slopes corresponding to the bidirectional attacks, shown by the blue diamonds, are generally close to -1, whereas the slopes corresponding to the unidirectional attacks, shown by the red circles, are close to zero. Moreover, the exact relation reported in the manuscript for the unidirectional case is

$$\left\langle \frac{df}{dc} \right\rangle^u = -\frac{1}{N},$$

where the superscripts u and b are used here to distinguish the unidirectional and bidirectional cases, respectively.

To further investigate the reviewer's observation, we revisited the first-order perturbation analysis for both types of attacks. Let

$$\boldsymbol{y}_0 := V\boldsymbol{v}_0 = \begin{bmatrix} y_{0,1} & \cdots & y_{0,N} & y_{0,N+1} \end{bmatrix}^\top$$

be the unit-norm effective eigenvector used in the perturbation analysis. Since $\boldsymbol{y}_0$ is in the range of V, it satisfies

$$\boldsymbol{y}_0^\top \boldsymbol{y}_0 = 1, \qquad \sum_{j=1}^{N+1} y_{0,j} = 0. \tag{1}$$

In particular,

$$\sum_{i=1}^{N} y_{0,i} = -y_{0,N+1}, \qquad \sum_{i=1}^{N} y_{0,i}^2 = 1 - y_{0,N+1}^2. \tag{2}$$

For a unidirectional attack on node i, first-order perturbation theory gives

$$\begin{aligned} \frac{df_i^u}{dc} &= \boldsymbol{y}_0^\top \frac{P_i^u + (P_i^u)^\top}{2} \boldsymbol{y}_0 \\ &= -y_{0,i}^2 + y_{0,i} y_{0,N+1}. \end{aligned} \tag{3}$$

Consequently, using Eq. (2),

$$\sum_{i=1}^{N} \frac{df_i^u}{dc} = -\sum_{i=1}^{N} y_{0,i}^2 + y_{0,N+1} \sum_{i=1}^{N} y_{0,i}$$
$$= -\left(1 - y_{0,N+1}^2\right) - y_{0,N+1}^2 = -1. \tag{4}$$

It therefore follows that

$$\left\langle \frac{df}{dc} \right\rangle^u := \frac{1}{N} \sum_{i=1}^{N} \frac{df_i^u}{dc} = -\frac{1}{N}. \tag{5}$$

Thus, the fact that the individual unidirectional slopes in Supplementary Fig. 5 are close to zero is consistent with the $-1/N$ relation. The scaling does not result from the individual unidirectional slopes being close to -1; rather, it follows from the topology-independent identity in Eq. (4).

For a bidirectional attack on node i, the corresponding perturbation matrix gives

$$\frac{df_i^b}{dc} = \boldsymbol{y}_0^\top \frac{P_i^b + (P_i^b)^\top}{2} \boldsymbol{y}_0$$
$$= -y_{0,i}^2 + 2y_{0,i} y_{0,N+1} - y_{0,N+1}^2$$
$$= -\left(y_{0,i} - y_{0,N+1}\right)^2. \tag{6}$$

Summing over all possible targeted nodes and using Eq. (2) yields

$$\sum_{i=1}^{N} \frac{df_i^b}{dc} = -\sum_{i=1}^{N} \left(y_{0,i} - y_{0,N+1}\right)^2$$
$$= -\sum_{i=1}^{N} y_{0,i}^2 + 2y_{0,N+1} \sum_{i=1}^{N} y_{0,i} - N y_{0,N+1}^2$$
$$= -\left[1 + (N+1) y_{0,N+1}^2\right]. \tag{7}$$

Therefore,

$$\left\langle \frac{df}{dc} \right\rangle^b := \frac{1}{N} \sum_{i=1}^{N} \frac{df_i^b}{dc} = -\frac{1 + (N+1) y_{0,N+1}^2}{N}. \tag{8}$$

Equation (8) identifies the reason that the bidirectional case does not exhibit the same universal scaling with N. In contrast to the unidirectional result, the bidirectional mean slope depends on the component $y_{0,N+1}$ of the perturbation eigenvector. This component is determined by the original network topology and is not, in general, a function of the network size alone.

One may formally write

$$\frac{\langle df/dc \rangle^b}{1 + (N+1) y_{0,N+1}^2} = -\frac{1}{N}. \tag{9}$$

However, the normalization factor in Eq. (9) is itself network dependent. Such a normalization would remove the topology-dependent part of the bidirectional response rather than reveal a universal scaling law based solely on the network size.

We therefore agree with the reviewer that the two cases exhibit consistent but distinct behaviors. The unidirectional result follows from a topology-independent identity and produces the universal relation $-1/N$. By contrast, the bidirectional result contains an additional topology-dependent eigenvector term. We were therefore unable to identify a topology-independent normalization, depending only on N, that makes the bidirectional results follow the same universal scaling law. We have included the above derivation and this clarification in Supplementary Note 4 of the revised Supplementary Information.

Comment Reviewer 3.2:

It is still not understandable why the standard deviation around 20-40 is the largest in SI figure 7. In the SI figure 6 bottom left, the Neural networks have the lowest $\min(df/dc)$ **and quite high average values. Then, the standard deviation may be larger, but it is not in SI Figure 7. To avioad any mistakes, I will suggest the authors carefully investigate these.**

Answer to Reviewer 3.2:
We thank the reviewer for raising this point. We have carefully re-examined the data and independently verified the calculations used to generate Supplementary Fig. 7. We did not identify any errors, and we are confident that the reported mean values and standard deviations are correct.

We agree that the variation in the standard deviation across different real-world networks is not immediately intuitive. While some networks, such as the neural networks shown in Supplementary Fig. 6, exhibit a relatively broad distribution of slopes, the overall standard deviation does not appear to follow a simple trend with either the minimum slope or the network size. At present, we do not have a theoretical explanation for why certain networks display larger standard deviations than others. Since this observation is not central to the main conclusions of the paper, we prefer not to speculate beyond what is supported by the data. We have nevertheless carefully verified that the numerical results reported in Supplementary Fig. 7 are correct.

Comment Reviewer 3.3:

Minor:
References [10] and [18] are the duplications.

Answer to Reviewer 3.3:
We thank the Reviewer for mentioning this. We have fixed the issue.
